# Cold Atmospheric Plasma‐Activated In Situ Hydrogel Induces Hair Regeneration Via Immune Microenvironment Remodeling

**DOI:** 10.1002/advs.202511962

**Published:** 2025-11-09

**Authors:** Jung Suk Kim, Junho Byun, Jaehyun Choi, Dongun Jin, Qiaoyun Li, Jaiwoo Lee, Han‐Gon Choi, Yu‐Kyoung Oh

**Affiliations:** ^1^ College of Pharmacy and Research Institute of Pharmaceutical Sciences Seoul National University Seoul 08826 Republic of Korea; ^2^ Research Institute of Pharmaceutical Sciences, College of Pharmacy Sookmyung Women's University Seoul 04310 Republic of Korea; ^3^ College of Pharmacy Korea University Sejong 30019 Republic of Korea; ^4^ College of Pharmacy Hanyang University Ansan 15588 Republic of Korea

**Keywords:** cold atmospheric plasma, hair follicle microenvironment, hair regeneration, in situ hydrogel, regulatory T cell

## Abstract

Hair loss is a prevalent condition with limited effective treatments. Here, a cold atmospheric plasma (CAP)‐activated in situ hydrogel is reported, which modulates the hair follicle immune microenvironment and promotes hair regeneration. CAP irradiation induces the gelation of a tyramine‐grafted hyaluronic acid solution containing interleukin‐2 (HTsol/IL2), forming a hydrogel (CAPgel/IL2) that retains CAP‐induced reactive oxygen species and IL2 within its matrix. In vivo, subcutaneous injection of HTsol/IL2 into the dorsal skin of mice, followed by CAP exposure at the injection site, induces in situ formation of CAPgel/IL2. In a depilated mouse model, CAPgel/IL2 treatment results in significantly greater hair regeneration than other groups, characterized by enlarged hair follicles and a thickened hypodermis. The prolonged retention of IL2 in CAPgel/IL2 leads to an increased expansion of Treg within the hair follicle microenvironment. Additionally, CAPgel/IL2‐treated mice exhibit accelerated skin pigmentation changes, indicating a transition from the telogen to the anagen phase. These findings suggest that CAPgel/IL2 enhances regulatory T cell expansion in the hair follicle microenvironment and may serve as a potential treatment for hair loss. Moreover, the CAP‐activated IL2 delivery strategy, which modulates skin immune responses, may be extended to other immunologic skin disorders.

## Graphical abstract

A cold atmospheric plasma‐activated in situ hydrogel (CAPgel/IL2) delivers reactive oxygen species and interleukin‐2 to modulate the hair follicle immune microenvironment, enhancing regulatory T‐cell expansion, dermal papilla cell proliferation, and the telogen‐to‐anagen transition for robust hair regeneration.

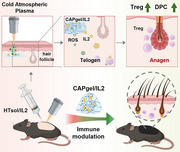

## 1 Introduction

Hair loss is a progressive disorder affecting millions worldwide, with significant psychological and quality‐of‐life impacts.^[^ ^1^ ^]^ Currently, the U.S. Food and Drug Administration has approved only two treatments for hair loss, minoxidil and finasteride.^[^ ^2^, ^3^ ^]^ While these drugs demonstrate therapeutic effects, they primarily alleviate symptoms rather than directly promoting hair growth, leaving an unmet need in hair loss treatment. Moreover, finasteride and minoxidil have been associated with side effects, including sexual dysfunction and an increased risk of prostate or breast cancer.^[^ ^4^ ^]^ Given these limitations, there is a growing demand for therapies that stimulate hair regeneration focusing on the hair follicle microenvironments.

Hair follicles are complex mini‐organs where stem and progenitor cells interact to regulate the local microenvironment.^[^ ^5^ ^]^ Hair growth is regulated by a cyclic process consisting of the anagen, catagen, and telogen phases, which recur throughout life.^[^ ^6^ ^]^ During the anagen phase, dermal papilla cells (DPCs) proliferate, stimulating hair regeneration, whereas in the telogen phase, they become quiescent.^[^ ^7^ ^]^ Hair loss occurs when a large proportion of hair follicles become arrested in the telogen phase and fail to transition into anagen due to biological or pathological factors. Notably, hair follicles remain inactive rather than disappearing.^[^ ^8^, ^9^ ^]^ Reactivating quiescent hair follicles and promoting their transition to a proliferative state is considered a promising strategy for hair loss treatment.

Regulatory T cells (Tregs) play a key role in regulating the immune microenvironment of hair follicles. The specialized CD4⁺ lymphocytes enforce immune tolerance and suppress excessive inflammation.^[^ ^10^, ^11^ ^]^ Tregs are abundantly present in the skin, where they regulate immune responses.^[^ ^12^ ^]^ Beyond their role in maintaining immune homeostasis, studies have shown that Treg contribute to skin regeneration, including hair follicle revitalization.^[^ ^13^, ^14^ ^]^ During the transition from telogen to anagen, Tregs accumulate around hair follicles and promote DPC proliferation.^[^ ^15^, ^16^, ^17^ ^]^ Based on these findings, increasing Treg and DPC populations in the hair follicle immune microenvironment may facilitate the telogen to anagen transition and serve as a potential strategy for hair loss treatment.

Interleukin‐2 (IL2) has been known to promote the expansion of Tregs. The α‐chain of the IL2 receptor is highly expressed in Tregs, making them more responsive to low IL2 concentrations than other immune cells.^[^ ^18^ ^]^ While Tregs primarily develop in the thymus, a subset arises from naïve CD4⁺ T cells in the periphery.^[^ ^19^ ^]^ Studies have shown that, in the absence of IL2, Tregs exhibit limited expansion and reduced survival in peripheral tissues.^[^ ^20^, ^21^ ^]^

In addition to IL2, reactive oxygen species (ROS) have also been shown to influence Treg expansion. As byproducts of mitochondrial energy production, ROS are inevitably generated as oxygen‐derived free radicals in aerobic organisms.^[^ ^22^ ^]^ Previous studies reported an association between ROS and oxidative stress, as well as cellular damage.^[^ ^23^ ^]^ However, recent findings indicate that low ROS levels play a beneficial role in normal signaling pathways, including the regulation of cell growth and T cell activation.^[^ ^24^, ^25^ ^]^ Moreover, ROS‐mediated redox regulation has been shown to facilitate Treg induction.^[^ ^26^ ^]^

Recent studies have investigated Treg‐mediated strategies for hair regeneration by using immunomodulatory biomaterials. Hexasaccharide fractions derived from oligosaccharides were shown to induce macrophages to selectively express chemokines, thereby recruiting Tregs into the hair follicle niche.^[^ ^27^ ^]^ In another study, an IL2–loaded microneedle system was designed to modulate the immune microenvironment and promote hair regeneration.^[^ ^28^ ^]^ However, IL2 alone produced only a minimal effect on altering the immune milieu, and co‐delivery with exogenous chemokines suffered from rapid dissipation at the injection site through diffusion and degradation, necessitating repeated administration.^[^ ^29^ ^]^ Moreover, chemokines primarily serve to recruit Tregs without directly promoting their proliferation.^[^ ^30^ ^]^ These limitations highlight the need for therapeutic systems that can achieve both recruitment and expansion of Tregs in a controlled and sustained manner.

In this study, we hypothesized that a cold atmospheric plasma (CAP)‐activated in situ hydrogel (CAPgel) containing interleukin‐2 (CAPgel/IL2) could modulate the hair follicle immune microenvironment by delivering reactive oxygen species (ROS) and IL2, thereby recruiting Tregs and simultaneously stimulating their selective expansion. CAP was employed as a ROS generator, while the hyaluronic acid‐tyramine (HT) conjugate functioned as a ROS‐responsive gel‐forming material. Upon ROS‐generating CAP irradiation, the HT solution (HTsol) containing IL2 (HTsol/IL2) undergoes gelation, forming CAPgel/IL2.

**Figure 1:**
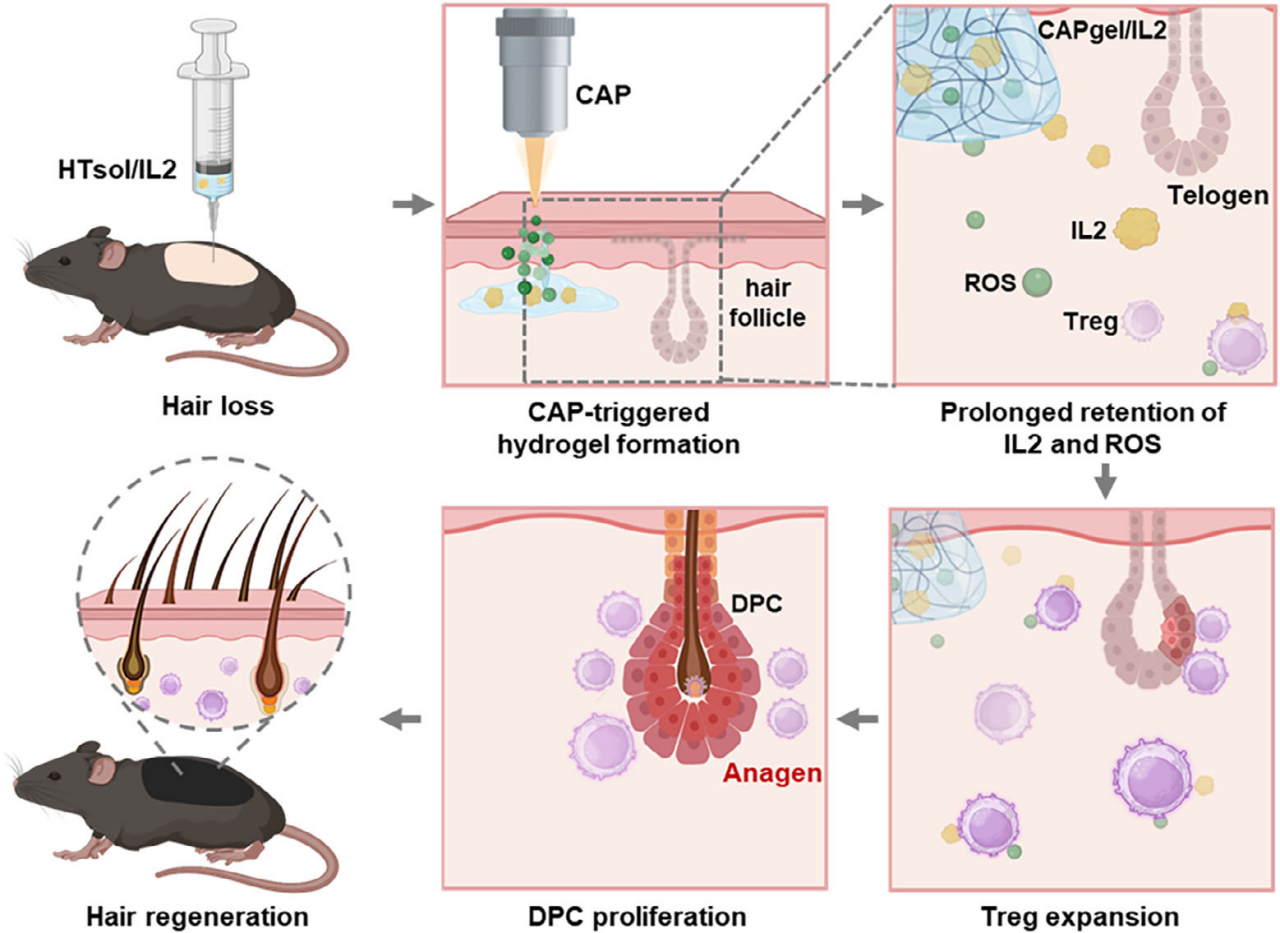
Proposed mechanism of CAP‐activated in situ hydrogel for inducing hair regeneration.

By combining localized IL2 release with ROS‐mediated hydrogel activation, CAPgel/IL2 provides a dual mechanism that overcomes the short‐lived effects of chemokines and enhances the efficiency of Treg modulation. Through this approach, we aimed to establish a more effective strategy for Treg‐driven hair regeneration with improved durability and translational potential (**Figure** **1**).

The mechanisms underlying CAPgel/IL2‐mediated hair regeneration are illustrated. Upon CAP irradiation, ROS generated by CAP can induce the in situ formation of CAPgel/IL2 from HTsol/IL2. The extended retention of IL2 and ROS within CAPgel/IL2 promotes the expansion of regulatory T cells, stimulates the proliferation of DPCs, accelerates the transition from the telogen to the anagen phase of hair follicles, and ultimately induces hair growth.

## 2 Results

### 2.1 Physicochemical Characteristics

HT synthesis, CAP‐induced gelation of HTsol/IL2, and the rheological properties of CAPgel/IL2 were characterized. In the HT conjugate, tyramine was grafted onto hyaluronic acid (HA) via an EDC/NHS‐mediated coupling reaction, as illustrated in Figure S1 (Supporting Information). The conjugation of tyramine to HA was confirmed by UV absorbance, ^1^H‐NMR, and FTIR spectroscopy. UV–vis spectroscopy revealed a strong absorption peak at 275 nm in HT, indicating the presence of the tyramine moiety (Figure S2, Supporting Information). Unlike HA, HT exhibited resonance peaks at 6.8 and 7.1 ppm, corresponding to the phenolic protons in tyramine moiety (Figure S3, Supporting Information). FTIR of HT showed a strong broad band at 3385 cm^−1^attributed to the stretching vibration of –OH groups, and 1550 cm^−1^, corresponding to amide bonds, respectively (Figure S4, Supporting Information).

**Figure 2:**
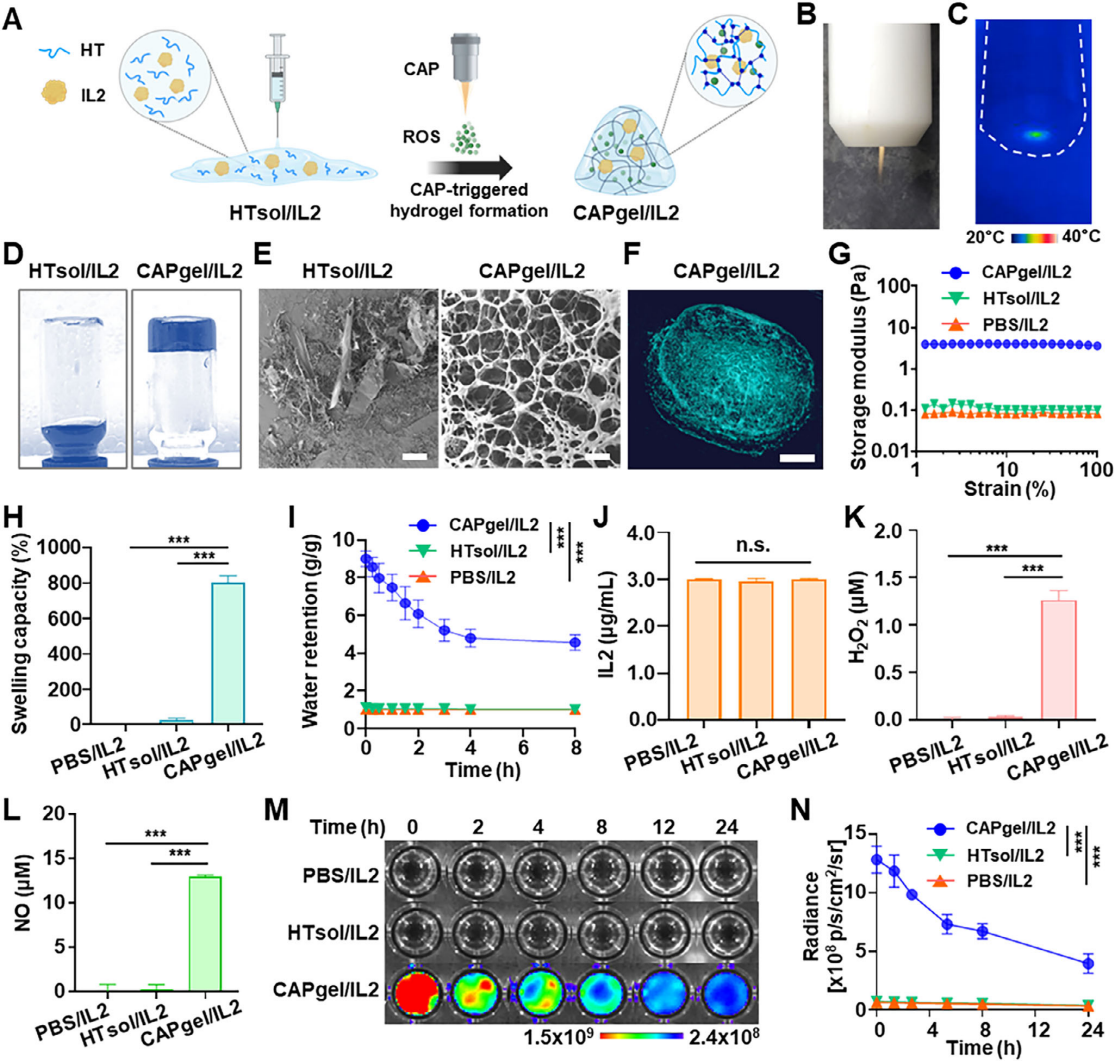
Physicochemical properties of CAPgel/IL2. A) A schematic illustration depicts the in situ gelation of CAPgel/IL2. B) The plasma jet is emitted from the CAP device. C) A thermal image captures plasma generated from the CAP device using an infrared thermal imaging camera. D) HTsol/IL2 and CAPgel/IL2 were stained with methylene blue and photographed. E) The surface morphology of CAPgel/IL2 was observed using SEM imaging (scale bar: 300 µm). F) The 3D structural morphology of CAPgel/IL2 was assessed using a Micro‐CT imaging system (scale bar: 0.25 cm). G) The rheological properties of CAPgel/IL2 were evaluated using a rotational rheometer. PBS/IL2 represents the group in which IL2 is dissolved in PBS at a concentration of 3.0 µg mL^−1^. H) The swelling capacity of CAPgel/IL2 was assessed after immersion in TDW (*n =* 3). I) The water retention capacity was analyzed over 24 h by incubating the water‐immersed formulations at 37 °C (*n =* 3). J) The amount of IL2 in the formulations was determined using an ELISA kit (*n =* 3). K,L) The quantification of ROS and nitric oxide was performed using the Amplex UltraRed assay (K) and the Griess reagent assay (L), respectively (*n =* 5). M) Fluorescence images displaying the ROS retention capacity were obtained using an IVIS Spectrum instrument. N) The quantified fluorescence intensity values of ROS are presented (*n =* 4). Statistical significance is indicated as ^***^ *p* < 0.001; n.s., not significant.

In situ gelation of HTsol/IL2 was induced by CAP, forming CAPgel/IL2 through a ROS‐mediated oxidative reaction (**Figure** **2**A). The plasma jet and CAP device used for gelation are shown in Figure 2B. Infrared thermal imaging measured the plasma temperature at 28.4 ± 0.3 °C during CAP operation (Figure 2C; Figure S5, Supporting Information). The formation of CAPgel/IL2 was observed in a vial inversion test, where CAPgel/IL2 remained intact and did not flow, unlike HTsol/IL2 (Figure 2D). Both CAPgel/IL2 and HTsol/IL2 were stained with methylene blue. SEM imaging showed that CAPgel/IL2 exhibited a porous, networked microstructure, distinct from the non‐crosslinked HTsol/IL2 (Figure 2E). Micro‐CT analysis visualized the 3D porous architecture of CAPgel/IL2 (Figure 2F), consistent with the SEM findings.

CAPgel/IL2 exhibited distinct rheological properties, swelling capacity, and water retention compared to other formulations. The storage moduli of CAPgel/IL2 were significantly higher than those of PBS/IL2 and HTsol/IL2 across the tested ranges (Figure 2G). CAPgel/IL2 showed the highest swelling capacity (800.4% ± 40.0%) among the tested formulations (Figure 2H). After 8 h of incubation at 37 °C, CAPgel/IL2 retained 4.5‐ and 4.6‐fold more water than HTsol/IL2 and PBS/IL2, respectively (Figure 2I). IL2 was released from CAPgel/IL2 in a sustained manner over 6 days (Figure S6A, Supporting Information). In parallel, the hydrogel underwent progressive degradation, with lyophilized mass decreasing to 66.4% of its initial weight by day 6 (Figure S6B, Supporting Information).

The levels and retention of reactive gas species varied significantly across formulations, whereas IL2 content remained similar. The IL2 concentration in CAPgel/IL2 was 2.99 ± 0.03 µg mL^−1^, with no significant difference from that of PBS/IL2 and HTsol/IL2 (Figure 2J). However, CAPgel/IL2 contained 37.2‐ and 52.7‐fold higher levels of ROS and nitric oxide compared to HTsol/IL2, respectively (Figure 2K,L). Fluorescence intensity measurements indicated that CAPgel/IL2 retained ROS, with detectable levels persisting up to 24 h at 25 °C (Figure 2M,N).

### 2.2 In Vitro Treg Expansion

**Figure 3:**
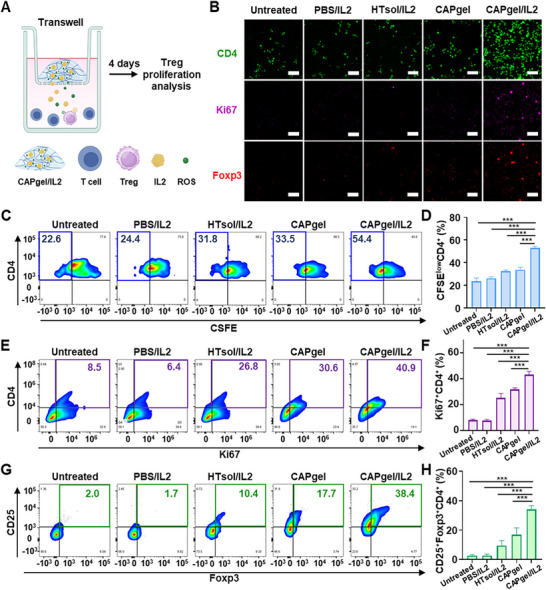
In vitro Treg expansion by CAPgel/IL2. A) The experimental scheme is illustrated. Splenic T cells were treated with various formulations using transwell inserts and incubated for 4 days. The T cells were then harvested and stained with relevant antibodies. B) The proliferation of Tregs was observed through confocal microscopy (scale bar: 30 µm). Tregs were stained with CD4 (green), Ki67 (purple), and Foxp3 (red). C,D) The populations of CFSE^low^ CD4^+^ T cells were plotted (C) and quantified (D) by flow cytometry. T cells were stained with CFSE at day 0 and underwent 4 days of incubation followed by staining with PE‐conjugated anti‐mouse CD4 antibody. E,F) The populations of Ki67⁺CD4⁺ T cells were plotted (E) and quantified (F) by flow cytometry. Proliferated CD4⁺ T cells were stained with an FITC‐conjugated anti‐mouse CD4 antibody, and intracellular staining was performed using Alexa Fluor 647‐conjugated anti‐mouse Ki67 antibody. G,H) The populations of CD25⁺Foxp3⁺CD4⁺ T cells were plotted (G) and quantified (H) by flow cytometry. T cells were first stained with a PE‐conjugated anti‐mouse CD4 antibody and an FITC‐conjugated anti‐mouse CD25 antibody, followed by intracellular staining with an APC‐conjugated anti‐mouse Foxp3 antibody. All statistical data are presented as mean ± SD (*n =* 5; ^***^ *p* < 0.001; n.s., not significant).

CAPgel/IL2 promoted significant Treg expansion compared to other formulations. Treg proliferation was assessed using confocal microscopy and flow cytometry. Splenic T cells from C57BL/6 mice were treated with various formulations using transwell inserts (**Figure** **3**A). After 4 days of incubation, Treg proliferation was visualized by confocal microscopy. Compared to the HTsol/IL2 and CAPgel groups, the CAPgel/IL2 group exhibited a greater expansion of Tregs, with increased populations of CD4⁺, Ki67⁺, and Foxp3⁺ cells (Figure 3B).

Flow cytometry analysis revealed that CAPgel/IL2 most effectively promoted Treg proliferation among the tested formulations. In the CFSE assay, cells with low CFSE signal intensity (CFSE^low^) were considered to be in a proliferative state. The CAPgel/IL2 group exhibited the highest population of CFSE^low^CD4⁺ T cells, with 2.0‐, 1.6‐, and 1.6‐fold increases compared to the PBS/IL2, HTsol/IL2, and CAPgel groups, respectively (Figure 3C,D). Similarly, the proportion of Ki67⁺CD4⁺ T cells in the CAPgel/IL2‐treated group was 5.3‐fold higher than that in the untreated group (Figure 3E,F). The CAPgel/IL2‐treated group also exhibited the highest population of CD25⁺Foxp3⁺CD4⁺ T cells among the evaluated groups (Figure 3G,H). Notably, CAPgel/IL2 treatment increased this population by 12.5‐, 3.6‐, and 2.0‐fold compared to PBS/IL2, HTsol/IL2, and CAPgel treatments, respectively. The gating strategy is shown in Figure S7 (Supporting Information).

CAP treatment significantly expanded proliferating and regulatory T cell populations in a ROS‐dependent manner. CAP treatment increased the CFSE^low^ CD4⁺ T cell population by 1.4‐fold and the CD25⁺Foxp3⁺CD4⁺ T cell population by 2.4‐fold compared with the untreated group. The addition of NAC after CAP treatment reduced the frequencies of both CFSE^low^ CD4⁺ T cells and CD25⁺Foxp3⁺CD4⁺ T cells to levels that were not significantly different from untreated controls (Figure S8, Supporting Information).

CAPgel/IL2 induced the strongest immunosuppressive cytokine response among the groups tested. ELISA results showed that treatment with CAP or IL2 alone increased IL10 and TGFβ production compared with the untreated group, whereas CAPgel/IL2 elicited the highest levels of these cytokines. The addition of NAC after CAP exposure significantly reduced cytokine production, resulting in little difference from the untreated group (Figure S9, Supporting Information).

IL2 exhibited a dose‐dependent effect, with 0.3 µg mL^−1^ representing an optimal concentration for preferential Treg expansion in vitro. Compared with untreated controls, both CD4⁺ and CD8⁺ T cells showed increased proportions of the CFSE^low^ population with rising IL2 concentrations (Figure S10A, Supporting Information). At 0.3 µg mL^−1^, the fold increase in CD4⁺ T cells relative to untreated cells exceeded that of CD8⁺ T cells, whereas at 3.0 µg mL^−1^, the fold increase in CD8⁺ T cells was greater than that of CD4⁺ T cells (Figure S10B, Supporting Information). Assessment of ROS‐mediated cytotoxicity showed that T cell viability was not significantly reduced up to 3 min of CAP exposure, a duration longer than that required for in situ hydrogel formation. In contrast, viability declined significantly after 5 min of exposure and decreased further at 10 min (Figure S11, Supporting Information).

### 2.3 Ex Vivo DPC Proliferation Upon Co‐Culture with T Cells

**Figure 4:**
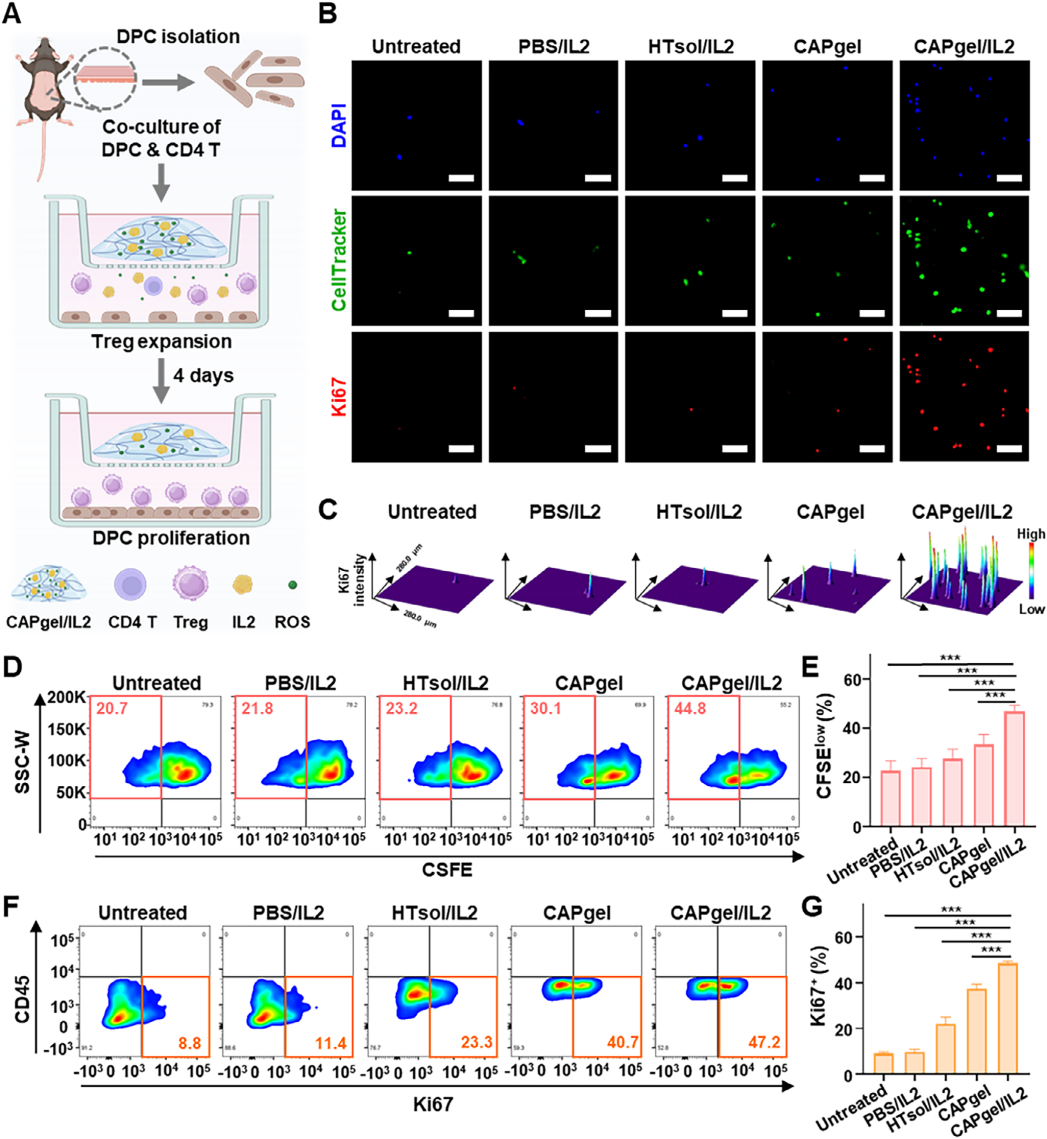
Ex vivo DPC proliferation in DPC and T cell co‐culture system. A) The ex vivo co‐culture study scheme is illustrated. The system consisted of DPCs and CD4⁺ T cells, where DPCs were collected from dorsal skin and CD4⁺ T cells were isolated from splenocytes. After 4 days of incubation with various formulations, the proliferation of DPCs was evaluated using fluorescence imaging and flow cytometry. B) The proliferation of DPCs was observed through THUNDER imaging (scale bar: 50 µm). On day 0, DPCs were pre‐labeled with CellTracker Green CMFDA dye and co‐cultured with CD4⁺ T cells. After 4 days of incubation with various formulations, intracellular staining was performed using Ki67 (red), and nuclei were stained with DAPI (blue). C) The 3D surface plots depict fluorescence intensity of Ki67 expression. Fluorescence images of Ki67‐expressing DPCs were converted into 3D surface plots using ImageJ software. D,E) The populations of CFSE^low^ DPC were plotted (D) and quantified (E) using flow cytometry. DPCs were pre‐stained with CFSE on day 0, and after 4 days of incubation with various formulations, surface staining was performed using a Cy5.5‐conjugated anti‐mouse CD45 antibody. F,G) The proportions of Ki67⁺ DPCs were plotted (F) and quantified (G) using flow cytometry. After 4 days of incubation with various formulations, surface staining was conducted using a Cy5.5‐conjugated anti‐mouse CD45 antibody, followed by intracellular staining with an Alexa Fluor 647‐conjugated anti‐mouse Ki67 antibody. All statistical data are presented as ^**^mean ± SD (*n =* 5; ^*^ *p* < 0.001).

Ex vivo treatment with CAPgel/IL2 significantly enhanced DPC proliferation. Co‐culture experiments were conducted as shown in **Figure** **4**A. After 4 days of incubation, DPC proliferation was analyzed using fluorescence imaging and flow cytometry. Fluorescence imaging with the THUNDER system revealed a marked increase in DPC proliferation in the CAPgel/IL2‐treated group compared to other groups (Figure 4B). The expression levels of Ki67, a proliferation marker, were the highest in the CAPgel/IL2‐treated group. 3D surface plots further showed the notable increase in Ki67 expression in the CAPgel/IL2‐treated group compared to groups treated with other formulations (Figure 4C).

Flow cytometry analysis showed that CAPgel/IL2 significantly increased the proportion of proliferating DPCs. The population of CFSE^low^ DPCs was higher in the CAPgel/IL2‐treated group than in other groups (Figure 4D,E). Specifically, CFSE^low^ DPCs in the CAPgel/IL2‐treated group were 1.7‐ and 1.4‐fold higher than in the HTsol/IL2‐ and CAPgel‐treated groups, respectively. CAPgel/IL2 also increased the proportion of Ki67⁺ DPCs (Figure 4F,G). In the CAPgel/IL2‐treated group, the Ki67⁺ DPC population was significantly higher than in groups treated with other formulations. The population of Ki67⁺ DPCs in the CAPgel/IL2‐treated group was 2.2‐ and 1.3‐fold higher than in the HTsol/IL2‐ and CAPgel‐treated groups, respectively.

### 2.4 In Vivo Retention of IL2, ROS, and CAPgel

**Figure 5:**
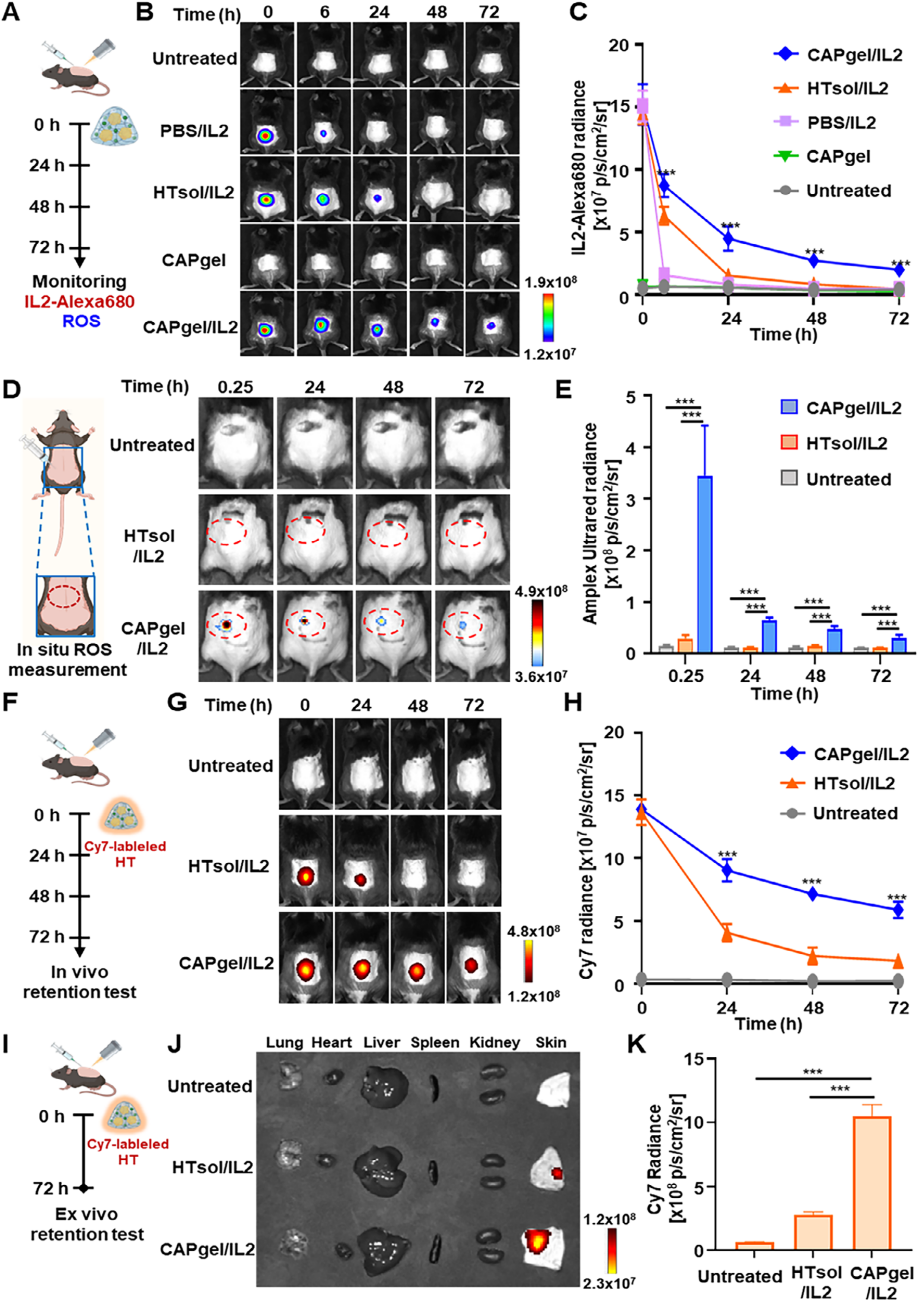
In vivo retention of IL2, ROS and CAPgel. A) The schedule for the in vivo retention study of IL2 and ROS is illustrated. After shaving the dorsal skin of C57BL/6 mice, HTsol/IL2 was subcutaneously injected into the skin and exposed to CAP. The retention of Alexa680‐labeled IL2 (IL2‐Alex680) and ROS at the injection site was monitored over 72 h using an IVIS Spectrum instrument. For fluorescence imaging, Alexa 680‐labeled IL2 and Amplex UltraRed were used for detecting IL2 and ROS, respectively. B) The retention of IL2 at the injection site was analyzed using fluorescence imaging. C) The fluorescence intensity of IL2 was quantified at each time point. D) The retention of ROS at the injection site was assessed using fluorescence imaging. E) The fluorescence intensity of ROS was quantified at each time point. F) The in vivo retention study schedule is illustrated. After shaving the dorsal skin of C57BL/6 mice, HTsol/IL2 was subcutaneously injected and exposed to CAP. CAPgel/IL2 retention was monitored over 72 h using an IVIS Spectrum instrument. For fluorescence imaging, Cy7‐labeled HT was used. G) Fluorescence imaging was used to analyze CAPgel/IL2 retention. H) Fluorescence intensity was quantified at each time point. I) The ex vivo hydrogel retention study schedule is illustrated. Cy7‐labeled HTsol/IL2 was subcutaneously injected into the shaved dorsal skin and irradiated with CAP. J) At 72 h post‐administration, dorsal skin tissue and vital organs, including the lungs, heart, liver, spleen, and kidneys, were extracted for ex vivo fluorescence imaging using the IVIS Spectrum instrument. K) Fluorescence intensities of dorsal skin tissue and vital organs were quantified. All statistical data are presented as mean ± SD (*n =* 5; ^*^ *p* < 0.001).

CAPgel/IL2 exhibited prolonged retention of IL2 compared to other formulations. To track IL2 retention, fluorescence dye‐labeled IL2 was used (**Figure** **5**A). After administering various formulations to mice, IL2 fluorescence intensity was monitored using imaging (Figure 5B,C). In mice treated with PBS/IL2, IL2 fluorescence intensity rapidly decreased within 6 h post‐dose. In the HTsol/IL2 group, IL2 retention was slightly extended, with detectable levels persisting for 24 h post‐dose. In contrast, the CAPgel/IL2 group maintained IL2 fluorescence, retaining 13.0% of the original signal at 72 h post‐administration, indicating extended IL2 retention.

CAPgel/IL2 significantly prolonged ROS retention at the injection site. ROS retention was evaluated by subcutaneously injecting a ROS‐detecting fluorescence agent at the injection site. The ROS levels in the HTsol/IL2 group did not significantly differ from those in the untreated group between 24 and 72 h post‐dose (Figure 5D,E). In contrast, the CAPgel/IL2 group exhibited significantly higher ROS levels at the injection site, with 11.7‐fold greater retention compared to the HTsol/IL2 group at 72 h post‐dose.

CAPgel/IL2 exhibited prolonged in vivo retention compared to HTsol/IL2. To assess hydrogel retention, Cy7‐labeled HT was used. Cy7‐HTsol/IL2 was subcutaneously injected into the shaved skin, followed by CAP irradiation for the CAPgel/IL2‐treated group (Figure 5F). After subcutaneous administration, HTsol/IL2 fluorescence disappeared by 24 h, whereas CAPgel/IL2 fluorescence persisted for up to 72 h (Figure 5G,H). Meanwhile, HTsol/IL2 subjected to 30 s of CAP exposure exhibited reduced in vivo retention compared with CAPgel/IL2 exposed for 150 s, but showed slightly prolonged persistence relative to unexposed HTsol/IL2 (Figure S12, Supporting Information). At 72 h post‐administration, major organs and dorsal skin tissues were harvested for ex vivo imaging (Figure 5I). Fluorescence intensity was significantly higher in the skin of CAPgel/IL2‐treated mice compared to the HTsol/IL2 group, but no significant differences were observed in major organs, including the lungs, heart, liver, spleen, and kidneys (Figure 5J; Figure S13, Supporting Information). The CAPgel/IL2 group exhibited greater Cy7‐labeled HT fluorescence than the HTsol/IL2 group (Figure 5J). At 72 h post‐dose, fluorescence intensity in the CAPgel/IL2‐treated group was 3.8‐fold higher than in the HTsol/IL2 group (Figure 5K).

### 2.5 Hair Regeneration in Depilated Mice

**Figure 6:**
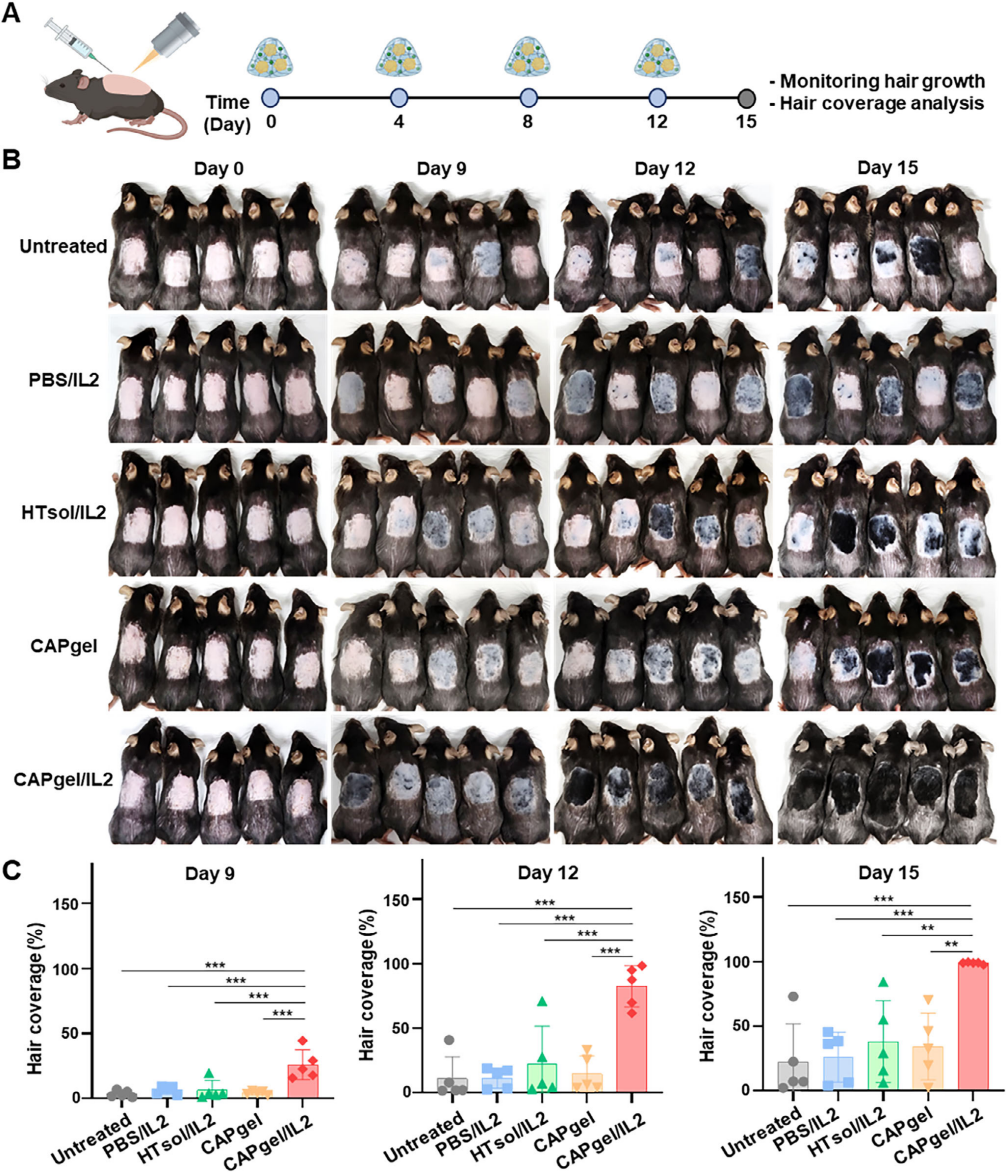
In vivo hair regeneration following treatments. A) A schematic illustration represents the in vivo hair regeneration study. The dorsal skin of 7‐week‐old mice was shaved, and various formulations were administered every 4 days for 2 weeks. B) Hair regeneration was monitored and imaged at each time point after the initial treatment. C) The percentage of hair coverage was quantified on days 9, 12, and 15 after the first treatment. All statistical data are presented as ^**^mean ± SD (*n =* 5; ^**^ *p* < 0.01, ^***^ *p* < 0.001).

CAPgel/IL2 accelerated hair regeneration in a depilated mouse model. Depilated mice were treated daily with various formulations for every 4 days, and hair regrowth was monitored (**Figure** **6**A). Hair regeneration efficacy was assessed through visual examination and hair coverage imaging analysis. By day 9, hair regrowth had initiated in CAPgel/IL2‐treated mice (Figure 6B). Unlike other groups, all CAPgel/IL2‐treated mice exhibited gray or blackish skin, indicating active pigmentation. By day 15, hair regeneration was clearly observed in all five CAPgel/IL2‐treated mice, whereas only partial hair regrowth was observed in the other groups.

CAPgel/IL2 treatment significantly enhanced hair regeneration, as quantified by hair coverage. At day 9, the hair coverage (%) in the CAPgel/IL2‐treated group was 7.5‐, 4.0‐, 4.1‐, and 6.0‐fold higher than in the untreated, PBS/IL2‐, HTsol/IL2‐, and CAPgel‐treated groups, respectively (Figure 6C). By day 15, hair coverage in the CAPgel/IL2‐treated group reached 99.0% ± 0.8%, a significantly higher value than in the other groups. Overall, the CAPgel/IL2‐treated group exhibited hair coverage that was 4.4‐, 3.8‐, 2.6‐, and 2.9‐fold higher than in the untreated, PBS/IL2‐, HTsol/IL2‐, and CAPgel‐treated groups, respectively. Notably, mice treated with CAPgel/IL2 showed smaller individual variations in hair coverage, whereas those receiving other formulations exhibited greater variability.

DPCs from CAPgel/IL2‐treated mice exhibited upregulation of the Wnt signaling pathway. Multiple Wnt ligands (Wnt2b/3a/4/5a/6/7a/8a/9a/10a/11) showed increased transcript levels across all three replicates, whereas Wnt antagonists (Dkk1, Sfrp1, Sfrp2) were decreased. At the intracellular signaling and transcriptional level, β‐catenin (Ctnnb1), the LEF/TCF transcriptional machinery (Tcf7, Tcf7l1, Lef1), and the co‐factor Ep300 were elevated. Canonical Wnt target genes associated with growth and matrix signaling (Myc, Jun, Ccnd1/2, Wisp1, Pitx2) were also upregulated (Figure S14, Supporting Information).

**Figure 7:**
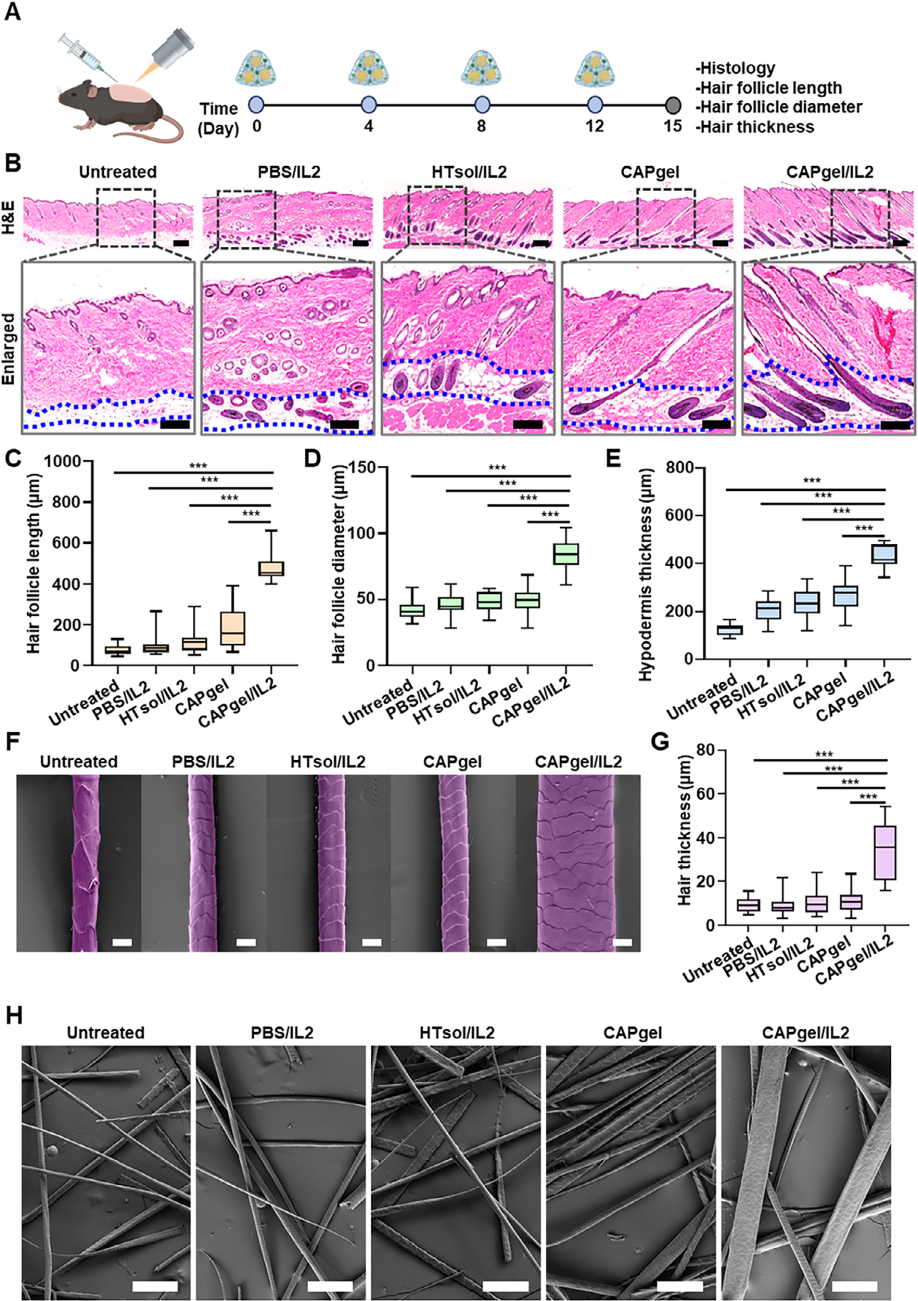
In vivo telogen to anagen transition of hair follicles. A) Depilated model mice were administered various formulations every 4 days on the shaved dorsal skin for 2 weeks. On day 15, the dorsal skin and regenerated hair were collected after sacrificing the mice for further experiments. B) Representative images of dorsal skin tissue stained with H&E are shown. The enlargement of hair follicles and hypodermis thickness were measured. The dotted line indicates the hypodermis (scale bar: 200 µm) C–E) Quantitative analyses were performed for hair follicle length (C, *n =* 25), hair follicle diameter (D, *n =* 25), and hypodermis thickness (E, *n =* 25). F) Representative pseudo‐colored SEM images of plucked hair at 15 days after the initial administration are displayed (scale bar: 10 µm). G) The quantitative analysis of hair thickness is presented (*n =* 25). H) Structure of randomly plucked hair analyzed by SEM imaging 15 days after the initial administration (scale bar: 100 µm). All statistical data are presented as ^**^mean ± SD (^*^ *p* < 0.001).

CAPgel/IL2 treatment increased hair follicle density and promoted elongation of hair follicles. Hair follicle morphology and hair thickness were analyzed on day 15 (**Figure** **7**A). Hematoxylin and eosin (H&E) staining revealed that hair follicles were scarcely observed in the untreated group (Figure 7B). The PBS/IL2‐ and HTsol/IL2‐treated groups exhibited a higher hair follicle density than the untreated group. The highest follicle density was observed in the hypodermis of the CAPgel/IL2‐treated group. Hair follicles in the CAPgel/IL2‐treated group exhibited a more elongated morphology compared to those in groups treated with other formulations.

CAPgel/IL2 treatment increased hair follicle length, diameter, and hypodermis thickness. The average hair follicle length in the CAPgel/IL2‐treated group was 481.0 ± 67.3 µm, which was 6.2‐, 5.2‐, 4.1‐, and 2.7‐fold higher than in the untreated, PBS/IL2‐, HTsol/IL2‐, and CAPgel‐treated groups, respectively (Figure 7C). Hair follicle diameter was also greater in the CAPgel/IL2‐treated group than in other groups, showing at least a 2.0‐fold increase (Figure 7D). Furthermore, hypodermal thickness and the number of hair follicles within the same skin area (tissue depth of 1300 µm) were significantly different in the CAPgel/IL2‐treated group compared with the other groups (Figure 7E; Figure S15, Supporting Information). Compared to the untreated group, the CAPgel/IL2‐treated group exhibited increased hair thickness (Figure 7F). The average hair thickness in the CAPgel/IL2‐treated mice was more than 3.0‐fold higher than in the untreated group or those treated with other formulations (Figure 7G). SEM imaging revealed that regrown hair in CAPgel/IL2‐treated mice appeared was thicker than in other groups (Figure 7H).

CAPgel/IL2 treatment did not induce abnormal inflammation or systemic toxicity. Thirty days after the first administration, H&E‐stained skin samples showed no inflammatory changes or tissue damage in the CAPgel/IL2‐treated group compared with untreated controls (Figure S16A, Supporting Information). Furthermore, serum analysis showed no significant differences in AST, ALT, BUN, or creatinine levels between CAPgel/IL2‐treated and untreated groups at either 15 or 30 days post‐treatment (Figure S16B, Supporting Information).

### 2.6 Modulation of Hair Follicle Immune Microenvironment

**Figure 8:**
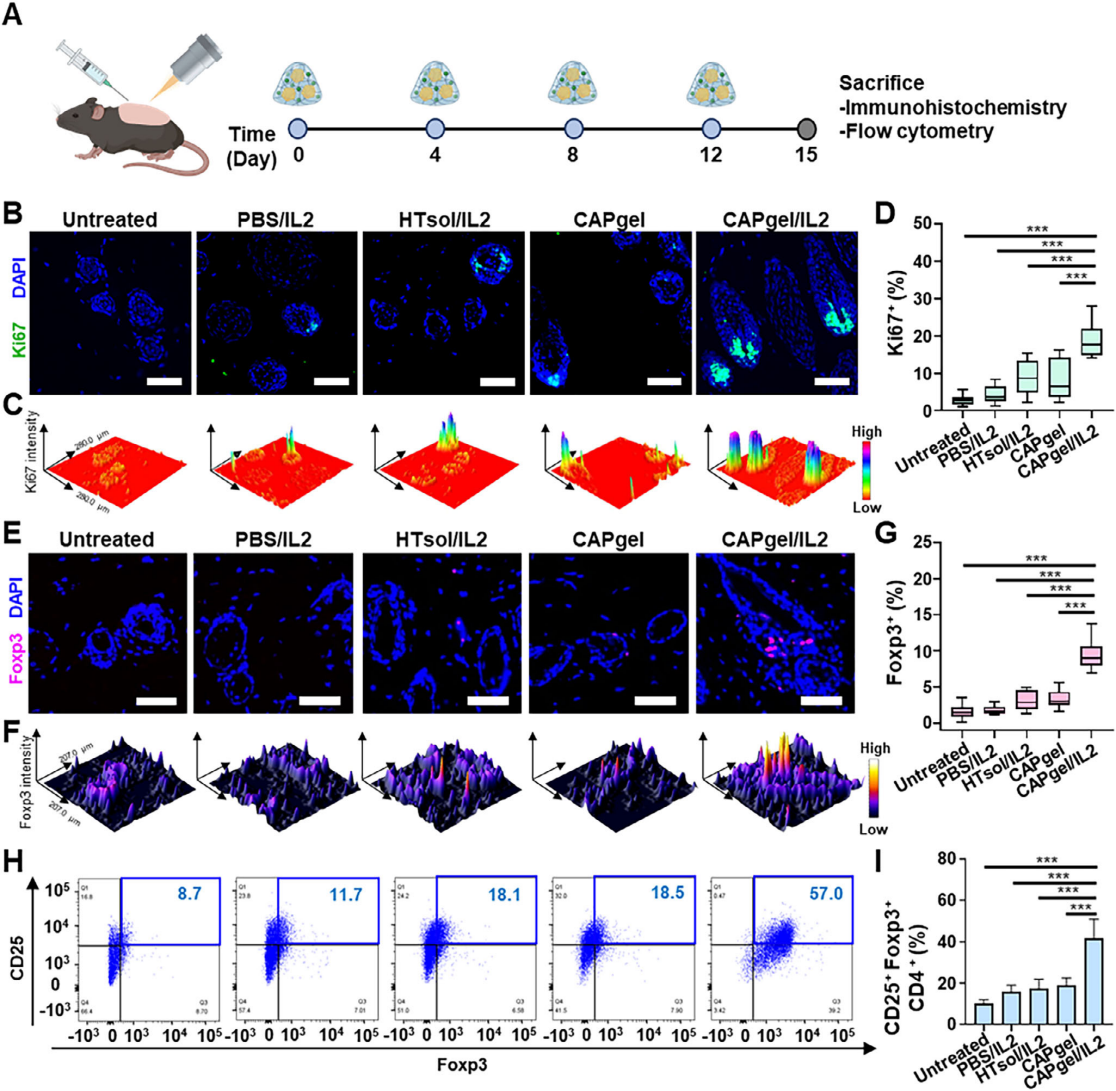
In vivo modulation of hair follicle immune microenvironment following treatments. A) Mice were treated four times with various formulations for every 4 days, and the dorsal skin was collected. For immunohistochemistry, the skin was stained with Alexa Fluor 647‐conjugated anti‐mouse Ki67 antibody and PE‐conjugated anti‐mouse Foxp3 antibody. For flow cytometry analysis, the skin tissue was digested to prepare a single‐cell suspension, followed by surface staining with BV605‐conjugated anti‐mouse CD3 antibody, APC/Cy7‐conjugated anti‐mouse CD4 antibody, and FITC‐conjugated anti‐mouse CD25 antibody. Intracellular staining was subsequently performed using a PE‐conjugated anti‐mouse Foxp3 antibody. B) The presence of Ki67‐positive cells in hair follicles was analyzed using the THUNDER imaging system (scale bar: 50 µm). The skin tissue was counterstained with DAPI (blue) and Ki67 (green). C) A 3D surface plot represents the fluorescence intensity of Ki67 in skin tissue. Immunofluorescence images were converted into 3D surface plots using ImageJ software. D) The proportions of Ki67⁺ cells in skin tissue were quantified based on immunohistochemistry results (*n =* 15). E) The presence of Tregs localized to hair follicles was analyzed using the THUNDER imaging system (scale bar: 50 µm). The skin tissue was counterstained with DAPI (blue) and Foxp3 (red). F) A 3D surface plot depicts the fluorescence intensity of Foxp3 in skin tissue. G) The proportions of Foxp3⁺ T cells in skin tissue were determined based on immunohistochemistry results (*n =* 15). H,I) CAPgel/IL2 increases the population of Tregs (CD25⁺Foxp3⁺CD4⁺ T cells) in skin tissue, as evaluated by flow cytometry. The populations of CD25⁺Foxp3⁺CD4⁺ T cells were plotted (H) and quantified (I) (*n =* 5). All statistical data are presented as ^**^mean ± SD (^*^ *p* < 0.001).

CAPgel/IL2 treatment increased Ki67⁺ cell populations in the hair follicle microenvironment. Ki67⁺ cell populations and regulatory T cells were analyzed using immunohistochemistry and flow cytometry (**Figure** **8**A). Immunohistochemical analysis revealed an increase in Ki67⁺ cells in the CAPgel/IL2‐treated group compared to other groups (Figure 8B). These Ki67⁺ cells were localized to the base of hair follicles in CAPgel/IL2‐treated mice. Additionally, 3D surface plots showed a clear contrast in Ki67 intensity between CAPgel/IL2‐treated mice and those treated with other formulations (Figure 8C). Quantitative analysis of immunohistochemistry images indicated that Ki67⁺ cell populations were significantly higher in the CAPgel/IL2‐treated group compared to other groups (Figure 8D).

CAPgel/IL2 treatment increased Treg populations near hair follicles. Fluorescence microscopy visualized the expansion of Tregs in CAPgel/IL2‐treated mice (Figure 8E). Tregs were localized in close proximity to hair follicles. 3D surface plot analysis showed a considerably higher Foxp3 intensity in the CAPgel/IL2‐treated group compared to other groups (Figure 8F). Image analysis indicated that Foxp3⁺ T cell populations were increased in the CAPgel/IL2‐treated group, with 5.9‐, 5.4‐, 3.0‐, and 2.8‐fold increases compared to the untreated, PBS/IL2‐, HTsol/IL2‐, and CAPgel‐treated groups, respectively (Figure 8G). Flow cytometry analysis showed that the Treg (CD25⁺Foxp3⁺CD4⁺ T cell) population was the highest in the CAPgel/IL2‐treated group (Figure 8H,I).

The regulatory T cell population in CAPgel/IL2‐treated mice increased by 4.1‐, 2.6‐, 2.4‐, and 2.2‐fold compared to the untreated, PBS/IL2‐, HTsol/IL2‐, and CAPgel‐treated groups, respectively. The gating strategy is shown in Figure S17 (Supporting Information). In addition, CAPgel/IL2 treatment altered the balance between regulatory and effector T cells in skin tissue. Flow cytometry analysis showed that CAPgel/IL2 treatment significantly increased the proportion of Tregs (CD25⁺Foxp3⁺). Meanwhile, the expansion of activated Teffs (CD25⁺Foxp3^−^) was minimal. The Treg‐to‐Teff ratio was elevated in CAPgel/IL2–treated skin, indicating selective immunomodulation (Figure S18, Supporting Information).

## 3 Discussion

CAPgel/IL2 expanded activated Tregs and promoted hair regeneration in a depilated mouse model. Following CAPgel/IL2, the prolonged retention of IL2 and ROS at the hair follicle microenvironment increased the proportion of activated Tregs. The modulated microenvironment with greater Treg populations led to DPC proliferation within hair follicles, ultimately enhancing hair regeneration.

Tregs play a crucial role in promoting the telogen‐to‐anagen transition in the hair regeneration cycle. Hair growth follows cyclic repetitions of the telogen, anagen, and catagen phases of hair follicles.^[^ ^31^ ^]^ Hair loss occurs when the transition from telogen (quiescent phase) to anagen (active phase) is impaired due to biological or pathological factors.^[^ ^5^ ^]^ To promote hair growth, stimulating the transition from telogen to anagen is essential. Recent studies have identified Tregs in the skin as key regulators of the hair regeneration cycle.^[^ ^12^ ^]^ During the late telogen phase, Tregs proliferate near hair follicles, facilitating the onset of anagen.^[^ ^16^ ^]^ Based on these findings, we developed CAPgel/IL2 to induce Treg proliferation in the hair follicle microenvironment, aiming to promote hair regeneration.

CAP‐generated ROS induced crosslinking reactions, leading to in situ gelation of HTsol/IL2. The ROS produced by CAP activated the generation of free radicals from the phenol groups in HT conjugates. These radicals facilitated coupling reactions, forming crosslinks either through C─C bonds between ortho‐carbons of the aromatic ring or C─O bonds between ortho‐carbons and phenolic oxygen.^[^ ^32^ ^]^ The formation of these crosslinks contributed to the in situ gelation of HTsol/IL2, resulting in CAPgel/IL2 (Figure 2A). The 3D microporous structure of CAPgel/IL2 was characterized by SEM imaging and micro‐CT analysis (Figure 2E,F). This porous structure is associated with high water absorption and retention capacity, enabling the hydrogel to exhibit swelling characteristics.^[^ ^33^ ^]^ In our experimental setting, CAP induced crosslinking between the tyramine groups of HTsol; therefore, hydrogel formation was only achievable in the presence of CAP, making the preparation of an IL2–loaded hydrogel without CAP impossible. As an alternative, we employed HTsol/IL2, an IL2–loaded non‐CAP hyaluronic acid–tyramine conjugate, to serve as a comparison group.

Comparison with previously reported hydrogel systems highlights the unique physicochemical features of the CAP‐induced hyaluronic acid hydrogel. Our hydrogel exhibited a highly porous network with pore diameters ranging from 100 to 600 µm, together with a relatively soft modulus, characteristics consistent with in situ gelation and efficient encapsulation of bioactive cargos (Table S1, Supporting Information). In contrast, a recent study reported that gelatin–alginate hydrogels crosslinked via polyethylene glycol diglycidyl ether and ionic interactions typically showed smaller pore dimensions (2–8 µm) and substantially higher stiffness (G′ up to 3 kPa).^[^ ^34^ ^]^ The larger pore size of our hydrogel enables accommodation of diverse therapeutic agents, including macromolecules and particulate carriers, while its compliant mechanical profile supports injectability and conformability to irregular tissue sites. Together, these properties distinguish the CAP‐induced hyaluronic acid hydrogel as a suitable in situ delivery platform, differing from conventional gelatin–alginate formulations that are primarily optimized for wound dressing applications.

Uniform gelation of CAPgel/IL2 requires precise irradiation by the CAP device, which presents challenges for scalability, clinical translation, and potential patient self‐administration. Most current CAP systems are designed for laboratory use and lack the portability needed for widespread clinical adoption. However, recent advances in compact CAP devices, including some that have already reached commercialization, suggest a feasible pathway for localized application in clinical or even home settings.^[^ ^35^ ^]^ Such portable systems may reduce barriers related to equipment size and accessibility, thereby broadening the translational potential of CAP‐based therapies.^[^ ^36^ ^]^ Future studies should evaluate the performance of these devices in preclinical and clinical contexts to determine their suitability for CAPgel/IL2 administration. In parallel, alternative ROS sources could provide complementary solutions for hydrogel crosslinking under more practical conditions. Inorganic peroxide compounds or photodynamic agents may serve as substitutes or adjuncts to CAP irradiation, potentially simplifying the process.^[^ ^37^ ^]^ Future work should investigate the efficiency, safety, and reproducibility of these alternatives compared with CAP‐based approaches. Integration of such methods with portable devices may further expand the range of clinically viable options for hydrogel activation. Taken together, these strategies may help address current technical limitations and support the advancement of CAPgel/IL2 toward clinical translation.

CAPgel/IL2 exhibited the highest in vitro efficacy in promoting Treg proliferation and activation. Ki67 and CD25 are widely used as markers of proliferating and activated regulatory T cells. CAPgel/IL2 treatment resulted in higher expression levels of Ki67 and CD25 compared to other groups (Figure 3). Ki67 serves as a cellular marker of proliferation, while CD25 represents the α‐chain of the heterotrimeric IL2 receptor, which is essential for Treg growth and activation.^[^ ^38^, ^39^ ^]^ Additionally, Ki67 expression has been linked to enlarged hair follicles.^[^ ^40^ ^]^ The increased Ki67 expression in the CAPgel/IL2‐treated group suggests that CAPgel/IL2 promoted anagen‐associated cell proliferation.

CAPgel/IL2 treatment prolonged ROS retention, which may have contributed to Treg expansion. ROS are highly reactive oxygen‐derived molecules generated through oxidation or reduction processes.^[^ ^41^ ^]^ Traditionally, ROS had been regarded as indicators of oxidative stress, leading to cellular damage. However, recent studies suggest that moderate ROS levels play a beneficial role in signaling pathways involved in T cell activation.^[^ ^42^ ^]^ In particular, reduced ROS levels have been associated with impaired regulatory T cell function, as ROS‐dependent metabolism influences Treg stability by modulating Foxp3 expression.^[^ ^43^ ^]^ The prolonged ROS retention observed in the CAPgel/IL2‐treated group may have contributed to the expansion of activated regulatory T cell populations.

Subcutaneous injection of HTsol/IL2 presents challenges for in situ gelation, as skin tissue may hinder ROS penetration from CAP. However, our data demonstrate that longer CAP exposure significantly improves depot persistence (Figure S12, Supporting Information). Although CAPgel/IL2 exhibited a relatively low storage modulus, its in vivo retention demonstrated sufficient stability for sustained localization. In retention studies, CAPgel/IL2 formed an in situ hydrogel upon CAP exposure and remained at the implantation site for more than 3 days (Figure 5H). In contrast, conventional subcutaneous implantation systems, which generally display higher storage moduli, require surgical administration and are often associated with adverse local inflammatory responses. CAPgel/IL2, despite its lower modulus, undergoes rapid gelation, persists in vivo for an adequate duration, and does not induce local tissue inflammation (Figure S16A, Supporting Information). These findings suggest that in vivo performance of CAPgel/IL2 is not strictly dependent on high storage modulus and that the formulation may help address limitations of conventional implantation systems.

CAPgel/IL2 treatment accelerated skin pigmentation, indicating early anagen transition. Skin pigmentation serves as a visible marker of the telogen‐to‐anagen transition in the hair cycle. As hair follicles progress from telogen (quiescent phase) to anagen (active phase), melanogenic activity of follicular melanocytes leads to skin pigmentation.^[^ ^44^ ^]^ Mice treated with CAPgel/IL2 exhibited earlier skin pigmentation compared to other groups. This suggests that CAPgel/IL2 promoted anagen progression to a greater extent than other formulations.

CAPgel/IL2 treatment induced anagen‐phase characteristics, including enlarged hair follicles and a thickened hypodermis. During the anagen phase, hair follicles expand and elongate, whereas they shrink during the telogen phase, as follicle size depends on the proliferative activity of cells within them.^[^ ^1^ ^]^ Additionally, hypodermal thickness increases as the hair cycle progresses from telogen to anagen. Increases in hypodermal thickness are closely associated with progression of the hair cycle from telogen to anagen. Hypodermal thickness has been reported to be directly linked to the process of hair follicle regeneration.^[^ ^45^ ^]^ Because hair follicles are deeply embedded within the hypodermal layer, active regeneration is typically accompanied by enlargement of follicular diameter, which can drive remodeling and thickening of the surrounding hypodermal structure.^[^ ^46^ ^]^ Expansion of the hypodermis also reflects the activation of dermal white adipose tissue, which has been implicated in supporting follicular stem cell activity and providing paracrine signals essential for anagen entry.^[^ ^47^ ^]^ Thus, measuring hypodermal thickness provides an indirect yet informative indicator of follicular growth, stem cell activation, and the transition into regenerative phases of the hair cycle. Assessing hypodermal thickness alongside direct measures of follicle size and density may offer a more comprehensive evaluation of hair regeneration dynamics.^[^ ^48^ ^]^ H&E staining further supported that CAPgel/IL2 promoted the telogen‐to‐anagen transition of hair follicles.

CAPgel/IL2 showed higher hair regeneration efficacy than FDA‐approved treatments in the depilated mice model. It has been reported that topical application of 1% minoxidil achieved under 40% hair coverage within 14 days.^[^ ^49^ ^]^ Another study showed that 5% minoxidil reached less than 50% coverage under similar conditions. In constrast to minoxidil, CAPgel/IL2 achieved complete (100%) coverage within 15 days.^[^ ^50^ ^]^ Importantly, CAPgel/IL2 required only four administrations at four‐day intervals, while minoxidil demanded daily application, indicating a more favorable dosing regimen. A comparable trend was observed with finasteride, as daily topical administration of 1% finasteride failed to induce significant hair regeneration compared with negative controls after 14 days.^[^ ^51^ ^]^ These results suggest that CAPgel/IL2 not only achieves greater efficacy but also reduces treatment frequency compared with standard therapies. Collectively, the data highlight the therapeutic potential of CAPgel/IL2 as an alternative strategy for hair regeneration, offering advantages in both efficacy and dosing convenience over existing FDA‐approved options.

Although the depilated mouse model has been widely used for assessing hair regeneration, it does not fully recapitulate the hormonal and genetic factors underlying human androgenetic alopecia, including androgen sensitivity of DPCs and progressive follicle miniaturization. To address this limitation, future studies should employ human‐derived systems that more closely mimic the disease context. Co‐culture models incorporating human DPCs and Tregs could be used to examine cellular interactions within the follicular niche, while ex vivo experiments with scalp hair follicles from patients with androgenetic alopecia would enable direct evaluation of follicle bulb thickening, anagen induction, and proliferation in response to CAPgel/IL2 treatment. In addition, the use of androgen‐stimulated organoid cultures or engineered follicle‐on‐a‐chip systems could provide controlled platforms to evaluate mechanistic responses to CAPgel/IL2 under conditions that mimic human androgenetic alopecia. Comparative studies with existing FDA‐approved therapies in these human‐relevant systems would also help assess the therapeutic advantages of CAPgel/IL2.

In addition to androgenetic alopecia, alopecia areata represents another major form of hair loss associated with substantial physical and psychological burden.^[^ ^52^ ^]^ This disorder is characterized by an autoimmune‐mediated attack on hair follicles, resulting in the collapse of immune privilege within the follicular niche.^[^ ^53^ ^]^ Dysregulated immune responses in alopecia areata involve autoreactive T cells together with impaired Treg function. C3H/HeJ mice are commonly used as a clinically relevant model of alopecia areata, in contrast to the depilation‐induced telogen model.^[^ ^54^ ^]^ Our results showed that CAPgel/IL2 significantly promoted hair regrowth even in the depilation‐induced model with a normal immune system. Given the central role of Treg dysfunction in alopecia areata, CAPgel/IL2 may exert stronger therapeutic effects in C3H/HeJ mice by restoring immune tolerance and promoting follicular regeneration under autoimmune conditions. Future studies employing this model would provide critical validation and enhance the translational relevance of CAPgel/IL2 for alopecia areata.

Long‐term histological analysis showed that CAPgel/IL2–treated mice maintained normal epidermal structure and collagen organization. Chronic skin inflammation is typically characterized by hemorrhage and marked infiltration of inflammatory immune cells.^[^ ^55^, ^56^ ^]^ In contrast, these features were absent in CAPgel/IL2–treated skin. Systemic biochemical evaluation also revealed no significant changes in hepatic or renal function markers, indicating the absence of systemic toxicity. These safety outcomes are consistent with the controlled release characteristics of CAPgel/IL2, which restrict ROS generation and IL2 delivery to the local tissue environment. Importantly, the absence of cumulative toxicity suggests that repeated administration may be feasible in chronic or regenerative treatment settings. Taken together, these findings suggest that CAPgel/IL2 induces minimal local or systemic toxicity and does not cause detectable oxidative damage from sustained ROS release, even after repeated administration and extended observation period.

The enhanced hair regeneration efficacy of CAPgel/IL2 over CAPgel alone or HTsol was associated with increased Treg populations in the hair follicle microenvironment. Flow cytometry analysis revealed that Treg populations were higher in the CAPgel/IL2‐treated group compared to the CAPgel‐treated group (Figure 3). IL2 is a cytokine that promotes T cell proliferation and differentiation.^[^ ^57^ ^]^ Low‐dose IL2 therapy has been shown to selectively expand regulatory T cells, as Tregs express a high‐affinity IL2 receptor, making them more responsive to IL2 compared to other T cells.^[^ ^58^ ^]^ Additionally, prolonged cytokine retention has been found to exert a greater effect on immune cells than low retention.^[^ ^59^ ^]^

CAPgel/IL2 modulated Treg responses and cytokine production, influencing the immune and regenerative microenvironment. CAPgel/IL2 treatment promoted Treg proliferation and increased secretion of IL10 and TGF‐β in vitro. The ROS dependency of this effect was demonstrated by the ability of NAC to abrogate CAP‐induced Treg expansion and cytokine production. CAP or IL2 alone induced modest increases in cytokine secretion, whereas their combined release from CAPgel/IL2 resulted in the highest levels, suggesting synergistic activity. In vivo, CAPgel/IL2 altered the immune microenvironment of DPCs through Treg expansion. Immunosuppressive cytokines including IL10 and TGF‐β have been reported to influence tissue regeneration.^[^ ^60^, ^61^ ^]^ The cytokine‐enriched environment generated by Treg expansion may contribute to the initiation of a pro‐regenerative transcriptional program in DPCs through the Wnt/β‐catenin pathway, which regulates hair cycle entry.^[^ ^31^, ^62^ ^]^ Collectively, the results suggest that CAPgel/IL2 may promote hair regeneration by integrating ROS and IL2 to modulate immune regulation for tissue repair.

Recent advances in bioengineered delivery systems, including biphasic microspheres, large‐scale bioengineered hair germs, and injectable microgels with exosomes, have demonstrated strategies to mimic the follicular niche and deliver growth factors in a spatiotemporally controlled manner.^[^ ^63^, ^64^, ^65^ ^]^ While these platforms primarily aim to recapitulate developmental signaling pathways, our CAPgel/IL2 system focuses on modulating the immune microenvironment by directly expanding Tregs. These two approaches are not mutually exclusive but may function in a complementary manner. A combined strategy that integrates Treg‐mediated immunoregulation with bioengineered constructs for growth factor delivery could simultaneously restore immune privilege, activate dermal papilla cells, and provide essential morphogenetic cues for follicle regeneration. Incorporating IL2 and ROS within such bioengineered scaffolds, alongside other growth factors, may enable coordinated regulation of both the immune and stem cell compartments of the hair follicle niche. Future exploration of this synergistic approach could extend the translational potential of CAPgel/IL2 and support the development of more effective and clinically relevant therapies for hair loss.

Although this study focused on the effect of CAPgel/IL2 on hair regeneration, its ability to modulate the immune microenvironment in the skin may have broader applications for immunological skin diseases. The prolonged retention of IL2 and ROS following CAPgel/IL2 treatment could be utilized to regulate local immune responses, potentially benefiting conditions associated with immune dysregulation, such as atopic dermatitis, psoriasis, and pemphigoid.^[^ ^66^, ^67^, ^68^, ^69^ ^]^ Given the crucial role of Tregs in maintaining immune homeostasis, CAPgel/IL2 may contribute to restoring immune balance in inflammatory and autoimmune skin disorders. Furthermore, biocompatible HA‐based CAPgel/IL2 could serve as a platform for localized cytokine delivery in cutaneous immunotherapy. Future studies are needed to further explore its therapeutic potential beyond hair regeneration, particularly in diseases where Treg modulation is a key treatment strategy.

## 4 Conclusion

In this study, we developed a CAP‐activated in situ hydrogel system to encapsulate IL2 and ROS within a hydrogel matrix for hair regeneration. Following CAPgel/IL2, the prolonged retention of IL2 and ROS in the hair follicle microenvironment enriched Tregs and stimulated DPC proliferation. The resulting immune modulation and cellular activation facilitated the telogen‐to‐anagen transition, effectively promoting hair regeneration. Overall, targeting Treg expansion to modulate the hair follicle immune microenvironment represents a promising therapeutic strategy for hair loss treatment.

## 5 Experimental Section

### Synthesis of HT Conjugate

Hyaluronic acid (HA) was conjugated with tyramine via a carbodiimide/active ester‐mediated coupling reaction, following a previously established method with slight modifications.^[^ ^70^ ^]^ Briefly, 100 mg of HA (201 kDa, 0.50 µmol; Contipro Inc., Dolní Dobrouč, Czech Republic) was fully dissolved in 10 mL of triple‐distilled water (TDW). Subsequently, 100 mg of 1‐ethyl‐3‐(3‐dimethylaminopropyl) carbodiimide (EDC) and 60 mg of N‐hydroxysuccinimide were added to the solution, adjusted to pH 4.7, and stirred for 3 h. Tyramine hydrochloride (225 mg, 1.325 mmol; Sigma–Aldrich, St. Louis, MO, USA) was then introduced and stirred overnight. The reaction mixture was dialyzed against 50% ethanol for 1 day, followed by TDW for an additional day (molecular weight cutoff: 2000 Da). The purified HT conjugate was lyophilized until further use. For preparation, the lyophilized HT powder (10 mg) was reconstituted in 1 mL of phosphate‐buffered saline (PBS, pH 7.4) to obtain a 50 µm HT solution. Before treatment, 100 mL of HT solution was mixed with freshly prepared 0.5 U of horseradish peroxidase to form HTsol, which was used for IL2 loading.

For some imaging experiments, HT was fluorescently labeled with cyanine 3 (Cy3) or cyanine 7 (Cy7). Briefly, 100 mg of HT was dissolved in 10 mL of TDW, followed by the addition of 100 mg of EDC and 60 mg of N‐hydroxysuccinimide. The solution was adjusted to pH 4.7 and stirred for 3 h. Subsequently, 0.525 µmol of Cy3‐amine or Cy7‐amine (Lumiprobe Corp, Hunt Valley, MD, USA) were introduced under continuous stirring, and the conjugation reaction proceeded overnight. The reaction mixture was dialyzed against 50% ethanol for 1 day, followed by TDW for an additional day (molecular weight cutoff: 2000 Da). The purified Cy3‐labeled HT (Cy3HT) or Cy7‐labeled HT (Cy7HT) were lyophilized and reconstituted in phosphate‐buffered saline (PBS, pH 7.4) at a concentration of 10 mg mL^−1^ for imaging experiments.

### Preparation of Hydrogel Loaded with IL2

CAP was used to induce the gelation of HTsol or HTsol/IL2. For HTsol/IL2 preparation, 0.3 µg of IL2 (BioLegend, San Diego, CA, USA) was added to 0.1 mL of HTsol (50 µm HT). A commercially available CAP device (FLA Medic+; FLAMME Inc., Icheon, Republic of Korea) was used to generate plasma under controlled conditions: compressed air flowed at a rate of 1 L min^−1^, and the applied voltage was 15 kV. The plasma temperature was monitored using an infrared thermal imaging system (FLIR T420; FLIR System Inc., Danderyd, Sweden).

HTsol and HTsol/IL2 were irradiated with CAP for 150 s to induce the formation of CAP‐induced hydrogels, CAPgel and CAPgel/IL2, respectively. Throughout CAP irradiation, the distance between the device and the solution was maintained at 1 cm. The resulting CAPgel was stored at 4 °C until use. To prepare CAPgel/IL2, 0.1 mL of HTsol (10 mg mL^−1^) containing IL2 (3.0 µg mL^−1^) was irradiated with CAP for 150 s. The IL2 content in CAPgel/IL2 was quantified using an IL2 enzyme‐linked immunosorbent assay (ELISA) kit (R&D Systems, Minneapolis, MN, USA).

### Spectroscopy and Morphology Studies

The structural features of HT conjugate were analyzed using UV spectroscopy, proton nuclear magnetic resonance (^1^H‐NMR), and Fourier‐transform infrared (FTIR) spectroscopy. UV absorbance was measured within the wavelength range of 240–360 nm using a UV–vis spectrometer (Orion AquaMate 8100; Thermo Fisher Scientific, Inc., Waltham, MA, USA). For ^1^H‐NMR analysis, samples were dissolved in deuterium oxide (Sigma–Aldrich) at 25 °C and analyzed using a Bruker Avance 400 NMR spectrometer (Bruker, Billerica, MA, USA). FTIR spectra were recorded using a JASCO 4700 spectrometer (JASCO, Tokyo, Japan) within the range of 4000–500 cm^−1^, with a resolution of 4 cm^−1^ per spectrum.

The morphology of HTsol/IL2 and CAPgel/IL2 was examined using scanning electron microscopy (SEM). Lyophilized CAPgel/IL2 or HTsol/IL2 samples were sputter‐coated with a 1.5 nm gold‐palladium layer before imaging. SEM analysis was performed using a field‐emission scanning electron microscope (Supra55VP; Carl Zeiss, Oberkochen, Germany). Additionally, CAPgel/IL2, labeled with a contrast agent (Angiofil; Medilumine Inc., Quebec, Canada), was visualized using a micro‐CT scanner (Quantum FX; PerkinElmer, Waltham, MA, USA) to assess the 3D structure of micropores within the hydrogel.

### Rheology and Swelling Capacity Tests

Rheological properties of the samples were evaluated using a rotational rheometer (ARES‐G2; TA Instruments, Ltd., New Castle, DE, USA). The frequency and the temperature of rheometer were set at 1 Hz and 25 °C, respectively. The swelling capacity of the samples was determined using a weighing method. The weight of the dehydrated formulation (W_d_) was measured, followed by immersion in water. Subsequently, the weight of the rehydrated formulation (W_s_) was measured. The swelling capacity was computed using the formula: swelling capacity (%) = (W_s_ ‐ W_d_) / W_d_ × 100. At predetermined time, the weight of the formulations was measured (W_t_). The water retention capacity was calculated with the following equation: water retention capacity = W_t_ / W_d_.

### In Vitro Release Study

To evaluate release behavior, CAPgel/IL2 was incubated in DMEM containing hyaluronidase (0.1 U mL^−1^) at 37 °C for 6 days.^[^ ^71^ ^]^ At predetermined time points (days 0–6), supernatants were collected and IL2 concentrations were quantified by ELISA (R&D Systems, Minneapolis, MN, USA) to determine cumulative release. At each time point, gels were retrieved, briefly incubated in TDW, frozen, and lyophilized mass was recorded.

### Analysis of ROS and Reactive Nitrite Species

For the analysis of ROS in the prepared formulations, hydrogen peroxide levels were measured using the Amplex UltraRed reagent (Thermo Fisher Scientific, Inc.), following the manufacturer's protocol. Briefly, 50 µL of 100 µm Amplex UltraRed reagent containing 0.2 U mL^−1^ of horseradish peroxidase was added to 250 µL of the formulations, followed by incubation in the dark for 30 min. Fluorescence intensity was measured using a SpectraMax M5 microplate reader (Molecular Devices, San Jose, CA, USA) with excitation and emission wavelengths of 530 and 590 nm, respectively. In certain experiments, the ROS retention capacity of the formulations was assessed using the Amplex UltraRed reagent. At specific time intervals, 20 µL of 100 µm Amplex UltraRed reagent containing 0.2 U mL^−1^ of horseradish peroxidase was added to 100 µL of the formulations. Fluorescence intensity was then measured using the IVIS Spectrum imaging system (PerkinElmer).

Reactive nitrite species levels in the formulations were quantified using the Griess reagent (Promega, Madison, WI, USA) according to the manufacturer's protocol. Briefly, 50 µL of sulfanilamide solution was mixed with 100 µL of the formulations and incubated in the dark for 10 min. Subsequently, 50 µL of N‐1‐naphthylethylenediamine dihydrochloride solution was added, followed by another 10 min incubation. Absorbance at 540 nm was measured using a SpectraMax M5 microplate reader, and nitrite concentrations were calculated.

### Animals

Seven‐week‐old C57BL/6 male mice were purchased from Raon Bio (Yongin, Republic of Korea) and housed under standard pathogen‐free conditions at the Animal Center for Pharmaceutical Research, Seoul National University. All animal studies were conducted in accordance with the Guidelines for the Care and Use of Laboratory Animals of the Institute of Laboratory Animal Resources, Seoul National University (approved protocol number: SNU‐220823‐1).

### Isolation and Culture of DPCs

DPC were isolated from C57BL/6 mice following a previously established method with slight modifications.^[^ ^72^ ^]^ Briefly, the dorsal skin of the mice was shaved and excised. After sterilization with 70% ethanol, the skin was digested in Roswell Park Memorial Institute 1640 (RPMI; Welgene, Gyeongsan, Republic of Korea) medium containing 0.2% type I collagenase (Sigma–Aldrich) at 37 °C for 2 h, then washed with PBS (pH 7.4).

Hair follicles were extracted from the dermis using a cell scraper and suspended in PBS (pH 7.4). The suspension was centrifuged at 1000 × g for 5 min, and the resulting hair follicle pellet was resuspended in Dulbecco's Modified Eagle Medium supplemented with 10% fetal bovine serum (GenDEPOT, Katy, TX, USA). The resuspended solution was mixed with an equal volume of 9% Ficoll solution and centrifuged at 200 × g for 5 min. After centrifugation, the isolated DPC were cultured in Dulbecco's Modified Eagle Medium supplemented with 10% fetal bovine serum.

### In Vitro Study of Treg Proliferation

Treg proliferation was assessed using a carboxyfluorescein succinimidyl ester (CFSE) assay and confocal imaging. T cells were isolated from splenocytes of C57BL/6 mice as previously described using a nylon column and seeded into a 24‐well plate at a density of 3 × 10⁵ cells per well in RPMI medium (Welgene) supplemented with 10% fetal bovine serum (GenDEPOT), 100 U mL^−1^ penicillin (Capricorn Scientific, Ebsdorfergrund, Germany), and 100 mg mL^−1^ streptomycin (Capricorn Scientific).^[^ ^73^ ^]^ T cells were treated with various formulations for 4 days using transwell inserts (SPL Life Sciences, Pocheon, Republic of Korea) with a diameter of 6.5 mm and a pore size of 0.4 µm. The culture medium surrounding the transwell inserts was replaced with fresh medium every 24 h. To evaluate ROS‐mediated effects, the ROS scavenger N‐acetyl‐L‐cysteine (NAC, Sigma–Aldrich) was added at a final concentration of 5 mm immediately after CAP treatment.

To perform flow cytometry analysis for CFSE assay and Foxp3 expression in Treg cell, splenocytes were pre‐stained with CFSE reagent (BioLegend) and treated with the prepared formulations. After incubation, the cells were harvested, and surface staining was performed using the following antibodies: phycoerythrin (PE)‐conjugated anti‐mouse CD4 antibody (1:100, Cat#116006, Lot#B370907, BioLegend), or FITC‐conjugated anti‐mouse CD25 antibody (1:100, Cat#102006, Lot#B252569, BioLegend). Dead cells were excluded using the Zombie NIR Fixable Viability Kit (BioLegend). For Ki67⁺CD4⁺ T cell measurement, a FITC‐conjugated anti‐mouse CD4 antibody (1:100, Cat#100406, Lot#B328710, BioLegend) was used for surface staining.

Intracellular staining was performed using a True‐Nuclear Transcription Factor Buffer Set (BioLegend). Briefly, after surface staining, cells were fixed with Fix Concentrate (BioLegend) for 1 h and washed with Perm Buffer (BioLegend). The intracellular components were then stained with Alexa Fluor 647‐conjugated anti‐mouse Ki67 antibody (1:100, Cat#151206, Lot#382946, BioLegend) or allophycocyanin (APC)‐conjugated anti‐mouse Foxp3 antibody (1:100, Cat#17‐5773‐82, Lot#1984797, Thermo Fisher Scientific, Inc.). Fluorescence intensity was detected using a BD FACS Lyric flow cytometry system (BD Biosciences, Franklin Lakes, NJ, USA).

For confocal imaging, Tregs were stained with FITC‐conjugated anti‐mouse CD4 antibody (1:100, Cat#100406, Lot#B328710, BioLegend), followed by fixation with 4% paraformaldehyde for 1 h. Intracellular staining was then performed using Perm Buffer (BioLegend) with PE‐conjugated anti‐mouse Foxp3 antibody (1:100, Cat#126404, Lot#B302809, BioLegend) and Alexa Fluor 647‐conjugated anti‐mouse Ki67 antibody (1:100, Cat#151206, Lot#382946, BioLegend). Nuclei were counterstained with 4′,6‐diamidino‐2‐phenylindole (DAPI, Sigma–Aldrich). Fluorescence signals were visualized using a Confocal Scope TCS8 (Leica, Wetzlar, Germany). The concentrations of the immunosuppressive cytokines IL10 and TGF‐β in the culture medium were measured using ELISA kits (TGF‐β ELISA, Cat. No. DY1679‐05; IL10 ELISA, Cat. No. DY417‐05; R&D Systems).

### In Vitro IL2 Titration Study

To assess the effects of IL2 on non‐Treg immune cell activation, a titration study was performed by varying IL2 concentrations. Collected T cells were seeded in 24‐well plates at equal cell numbers in identical culture media and treated with three different IL2 concentrations (0.3, 1.5, and 3.0 µg mL^−1^). Proliferation of CD4⁺ and CD8⁺ T cells was then analyzed using a CFSE assay as previously described.

### In Vitro Cytotoxicity Study

To evaluate the potential cytotoxic effects of ROS generated by CAP on T cells, cell viability was examined under different exposure conditions. Collected T cells were seeded in 24‐well plates at equal cell numbers in identical culture media. After CAP exposure for 3, 5, or 10 min, cell viability was measured using the 3‐(4,5‐dimethylthiazol‐2‐yl)‐2,5‐diphenyltetrazolium bromide (MTT) assay (Alfa Aesar, Haverhill, MA, USA; cat. no. L11939).

### In Vitro DPC Proliferation Study

The effect of various formulations on DPC proliferation after co‐culture with CD4⁺ T cells was evaluated using flow cytometry and fluorescence imaging. CD4⁺ T cells were isolated from splenocytes using a mouse CD4⁺ T cell isolation kit (MojoSort; BioLegend). For fluorescence imaging, DPCs were pre‐labeled with CellTracker Green CMFDA Dye (Thermo Fisher Scientific, Inc.), while for CFSE proliferation assays, they were pre‐stained with CFSE (BioLegend). DPCs and CD4⁺ T cells were seeded at densities of 5 × 10⁵ and 2 × 10⁵ cells per well, respectively. Various formulations were applied to transwell inserts (SPL Life Sciences, Pocheon, Republic of Korea), and the culture medium surrounding the inserts was replaced with fresh medium every 24 h.

For flow cytometry, DPCs and CD4⁺ T cells were subjected to surface staining with and Peridinin‐chlorophyll‐protein complex (PerCP)/Cy5.5‐conjugated anti‐mouse CD45 antibody (1:100, Cat#103132, Lot#B370638, BioLegend), and the FITC‐conjugated anti‐mouse CD4 antibody (1:100, Cat#100406, Lot#B328710, BioLegend), respectively.

For flow cytometry, DPCs and CD4⁺ T cells were harvested and subjected to surface staining with the following antibodies: FITC‐conjugated anti‐mouse CD4 antibody (1:100, Cat#100406, Lot#B328710, BioLegend) and peridinin‐chlorophyll‐protein complex (PerCP)/Cy5.5‐conjugated anti‐mouse CD45 antibody (1:100, Cat#103132, Lot#B370638, BioLegend). Cells positive with CD4 and CD45 were sorted as CD4⁺ T cells. Cells negative with CD45 were sorted as DPCs.

Dead cells were excluded using the Zombie NIR Fixable Viability Kit (BioLegend). Intracellular staining was performed using a True‐Nuclear Transcription Factor Buffer Set (BioLegend) with Alexa Fluor 647‐conjugated anti‐mouse Ki67 antibody (1:100, Cat#151206, Lot#382946, BioLegend). Ki67 expression in DPCs was analyzed using a BD FACS Lyric flow cytometer (BD Biosciences, Franklin Lakes, NJ, USA).

For fluorescence imaging, cells were fixed with 4% paraformaldehyde for 1 h. Intracellular staining was then performed using Perm Buffer (BioLegend) with Alexa Fluor 647‐conjugated anti‐mouse Ki67 antibody, and nuclei were counterstained with DAPI (Sigma–Aldrich). Fluorescence signals were visualized using a THUNDER imaging system (Leica).

### In Vivo Retention Study of IL2, ROS, and CAPgel

After subcutaneous injection of C57BL/6 mice with various formulations, the in vivo retention of IL2, ROS, and CAPgel was assessed using the IVIS Spectrum in vivo Imaging System (PerkinElmer). To evaluate CAPgel retention, mice were treated with 0.1 mL of Cy7HTsol/IL2 containing 0.3 µg IL2 and irradiated with CAP for 150 s. Alexa Fluor 680‐labeled IL2, Amplex UltraRed reagent, and Cy7‐labeled HT were used to detect CAPgel.

For ROS level assessment, 20 µL of 100 µm Amplex UltraRed reagent containing 0.2 U mL^−1^ of horseradish peroxidase was subcutaneously pre‐injected at the sample injection site before each measurement. The fluorescence intensity of Amplex UltraRed was used to detect ROS. Fluorescence intensity of Alexa Fluor 680‐labeled IL2, Amplex UltraRed for ROS, and Cy7‐labeled HT was monitored at predetermined time intervals using the IVIS Spectrum in vivo Imaging System (PerkinElmer).

### In Vivo Assessment of Hair Regeneration

Male 7‐week‐old C57BL/6 mice, whose hair follicles were in the telogen phase, were used for the in vivo efficacy study. Dorsal hair was removed using a depilatory cream. Mice were subcutaneously injected with various formulations and irradiated with CAP to induce in situ gelation. For the CAPgel/IL2 group, mice received a subcutaneous injection of 100 µL HTsol/IL2 containing 1 mg HT and 0.3 µg IL2, followed by 150 s of CAP irradiation. Treatments were administered for every 4 days. Hair growth in the treated skin area was monitored throughout the study, and hair coverage (%) was quantified using image analysis software (ImageJ, Version 1.54i, National Institutes of Health, USA).

Mice were sacrificed 15 days after the first treatment. Dorsal skin tissues were collected, fixed in 10% formalin, and embedded in paraffin. H&E staining was performed, and images were captured using the Vectra 3.0 Automated Quantitative Pathology Imaging System (PerkinElmer). For SEM imaging, revitalized hair was collected before sacrifice, sputter‐coated with a 1.5 nm gold‐palladium layer, and analyzed using a field‐emission scanning electron microscope (Supra55VP; Carl Zeiss).

To analyze the gene expression profiles associated with the Wnt signaling pathway, DPCs were isolated from the dorsal skin of mice following sacrifice. Total RNA was extracted from the collected DPCs, and complementary DNA (cDNA) was synthesized using Maxime RT PreMix (iNtRON Biotechnology, Seongnam, Republic of Korea; Cat. No. 25081). Quantitative PCR was subsequently performed with the AccuTarget qPCR Screening Kit (Bioneer, Daejeon, Republic of Korea). Gene expression levels were normalized to GAPDH, and the log_10_ fold changes were calculated relative to untreated DPCs.

For long‐term skin safety evaluation, mice were sacrificed 30 days after the first treatment, and dorsal skin tissues were collected and processed using the same procedure. To assess systemic toxicity of CAPgel/IL2, serum samples were collected at the same time point and analyzed for alanine transaminase (ALT), aspartate transaminase (AST), blood urea nitrogen (BUN), and creatinine levels.

### Immunohistochemistry of Skin Microenvironments

The proliferation of DPCs and Tregs in the treated skin microenvironment was assessed using immunohistochemistry. Tissues were deparaffinized, rehydrated, and subjected to heat‐induced antigen retrieval with Target Retrieval Solution (pH 6.0) (Agilent Dako, Santa Clara, CA, USA). The slides were then permeabilized with 0.1% Triton X‐100 and blocked with 10% goat serum in PBS for 1 h.

For staining, the slides were incubated overnight at 4 °C with Alexa Fluor 647‐conjugated anti‐mouse Ki67 antibody (1:100, Cat#151206, Lot#B382946, BioLegend) and PE‐conjugated anti‐mouse Foxp3 antibody (1:100, Cat#126404, Lot#B302809, BioLegend). Tissues were counterstained with DAPI (Sigma–Aldrich) and imaged using the THUNDER Imager 3D Assay (Leica). Quantitative image analysis was performed using InForm 2.2.1 image analysis software (PerkinElmer).

### Flow Cytometry of Tregs in Skin Microenvironments

The population of Tregs in the skin microenvironment was assessed using flow cytometry. Mice were sacrificed 15 days after the first treatment, and the dorsal skin was harvested and gently defatted. A single‐cell suspension was prepared following a previously established method with slight modifications.^[^ ^38^ ^]^ The skin was finely minced with scissors and suspended in RPMI medium supplemented with collagenase (2 mg mL^−1^), hyaluronidase (0.5 mg mL^−1^, Sigma–Aldrich), and DNase (0.1 mg mL^−1^, Sigma–Aldrich). After 1 h of incubation at 37 °C, the suspension was filtered through a cell strainer and centrifuged at 800 × g for 3 min.

For surface antigen staining, cells were labeled with Brilliant Violet 605 (BV605)‐conjugated anti‐mouse CD3 antibody (1:100, Cat#100237, Lot#B452856), APC/Cy7‐conjugated anti‐mouse CD4 antibody (1:100, Cat#100414, Lot#B326190), and FITC‐conjugated anti‐mouse CD25 antibody (1:100, Cat#102006, Lot#B252569). Intracellular staining was then performed using a True‐Nuclear Transcription Factor Buffer Set (BioLegend) with PE‐conjugated anti‐mouse Foxp3 antibody (1:100, Cat#126404, Lot#B302809). Treg populations were analyzed using an LSRFortessa X‐20 cell analyzer (BD Biosciences).

### Statistical Analysis

Statistical analysis of multiple groups was performed using one‐way analysis of variance (ANOVA) followed by Tukey's post‐hoc test for multiple group comparisons. For comparisons between two groups, a two‐tailed Student's *t*‐test was used. All statistical analyses were conducted using GraphPad Prism software (v8.0, GraphPad Software, San Diego, CA, USA). A *p*‐value of less than 0.05 was considered statistically significant.

## Conflict of Interest

The authors declare no conflict of interest.

## Supporting information



Supporting Information

## Data Availability

The data that support the findings of this study are available from the corresponding author upon reasonable request.
