# Supplementary material for: Cold Atmospheric Plasma‐Activated In Situ Hydrogel Induces Hair Regeneration Via Immune Microenvironment Remodeling
Source: Adv Sci (Weinh). 2025 Nov 9;13(5):e11962. doi: 10.1002/advs.202511962 (PMC12850053; doi:10.1002/advs.202511962)
Supplement: Supplementary file 1 — Supporting Information [file ADVS-13-e11962-s001.docx]

**Supplementary Table 1. Comparison of physicochemical features of hydrogels.**

| Hydrogel System | Crosslinking Method | Pore size | Stiffness metric | Drug-delivery context | Reference |
| --- | --- | --- | --- | --- | --- |
| Hyaluronic acid hydrogels | CAP-induced crosslinking | 100–600 µm (SEM) | Storage modulus G′: 7 Pa | In situ hydrogel | Used in this study |
| Gelatin–Alginate hydrogels | Combined covalent and ionic crosslinking | 2–8 µm (SEM) | Storage modulus G′ up to ~3 kPa | Wound dressing | [34] |

**Supplementary figures**

**
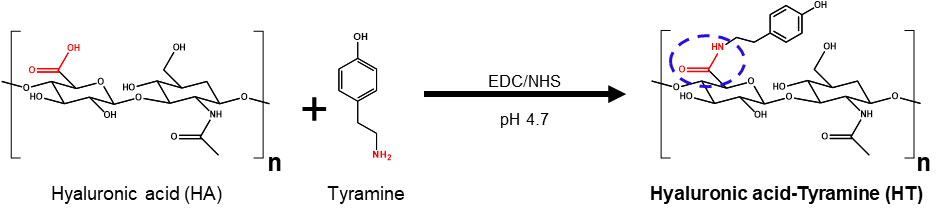
**

**Fig. S1. Schematic illustration of HT synthesis.**

The synthesis of HT was carried out using a carbodiimide/active ester-mediated coupling reaction.


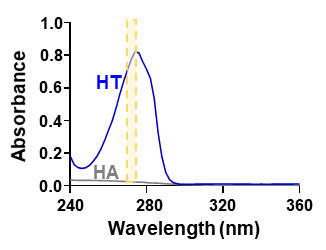


**Fig. S2. UV-VIS spectra of HA and HT.**

HA and HT were dissolved in TDW and analyzed using a UV-VIS spectrometer within the wavelength range of 240-360 nm.


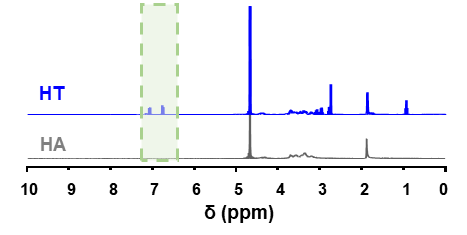


**Fig. S3. ^1^H-NMR spectra of HT and HA.**

HA and HT were dissolved in deuterium oxide at 25 °C and analyzed using an NMR spectrometer (400 MHz).


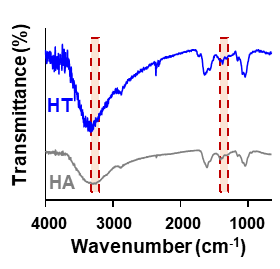


**Fig. S4. FTIR spectra of lyophilized HA and HT.**

The FTIR spectra of lyophilized HA and HT were analyzed within the range of 4,000–500 cm⁻¹ with a spectral resolution of 4 cm⁻¹.


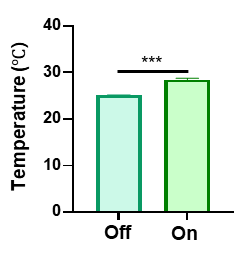


**Fig. S5. Temperature of the plasma generated from CAP device.**

The temperature of the plasma generated by the CAP device was analyzed using a thermal camera while the device was on and compared to the temperature when the device was off. Data are presented as mean ± SD (*n =* 3; ***p < 0.001, Student’s t-test).


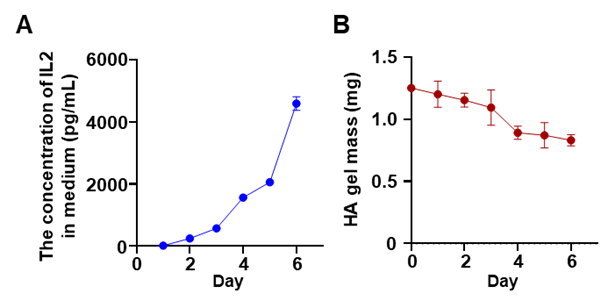


**Fig. S6. Degradation and IL2 release from CAPgel/IL2.**

(A) The cumulative release of IL2 was quantified by ELISA over 6 days (*n =* 5). (B) Hydrogel degradation was evaluated by measuring the lyophilized dry mass at the indicated time points (*n =* 5). All data are presented as mean ± SD.


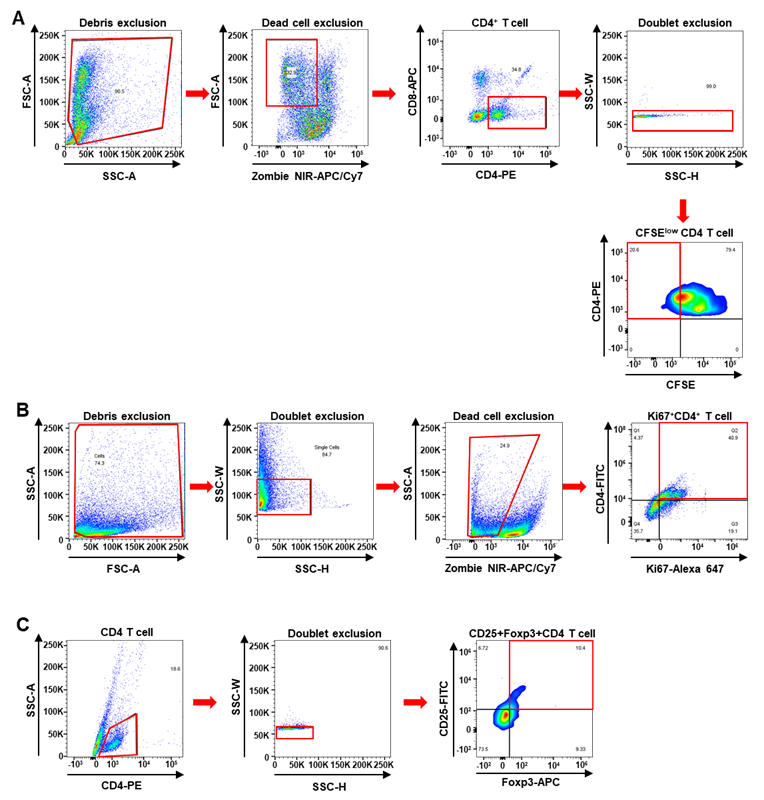


**Fig. S7. Gating strategy for in vitro T cell FACS analysis.**

(A) CFSElow CD4+ T cells plotted in Fig.3C were gated from CD4+CD8- T cells obtained from singlet suspensions of T cells after dead cell exclusion. (B) Ki67⁺CD4⁺ T cells plotted in Fig.3E were gated from singlet suspension of T cells after dead cell exclusion. (C) CD25+Foxp3+ Treg cells plotted in Fig.3G were gated from single suspensions of CD4 T cells.


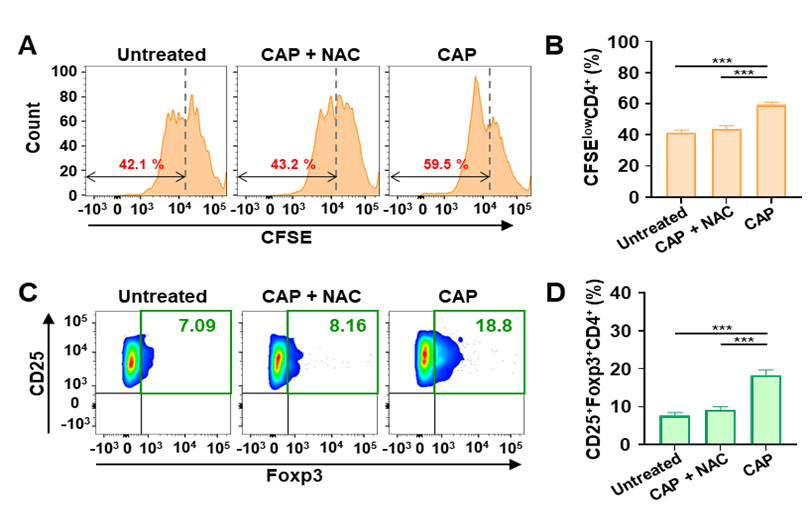


**Fig. S8. In vitro Treg expansion by ROS from CAP irradiation.**

Splenic T cells were exposed to CAP in the absence or presence of the ROS scavenger NAC. (A, B) The populations of CFSElow CD4⁺ T cells were plotted (A) and quantified (B) by flow cytometry. The T cells were stained with CFSE at day 0, incubated for 4 days, and then stained with a PE-conjugated anti-mouse CD4 antibody. (C, D) The populations of CD25⁺Foxp3⁺CD4⁺ T cells were plotted (C) and quantified (D) by flow cytometry. The T cells were first stained with a PE-conjugated anti-mouse CD4 antibody and an FITC-conjugated anti-mouse CD25 antibody, followed by intracellular staining with an APC-conjugated anti-mouse Foxp3 antibody. All statistical data are presented as mean ± SD (*n =* 5; ***p < 0.001).


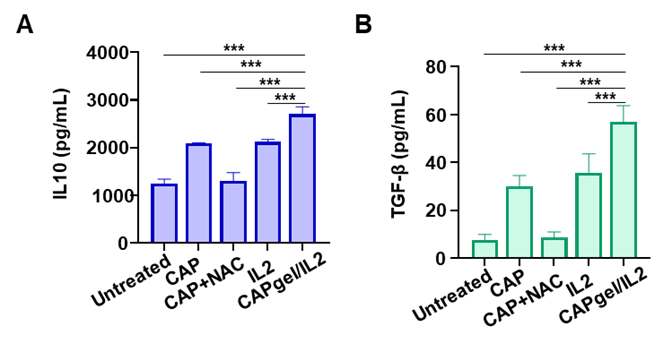


**Fig. S9. Induction of immunosuppressive cytokines following various treatments.**

Splenic T cells were treated with different formulations in transwell inserts and incubated for 4 days. The levels of the immunosuppressive cytokines IL10 (A) and TGF-β (B) were quantified by ELISA. All statistical data are presented as mean ± SD (*n =* 3; ***p < 0.001).


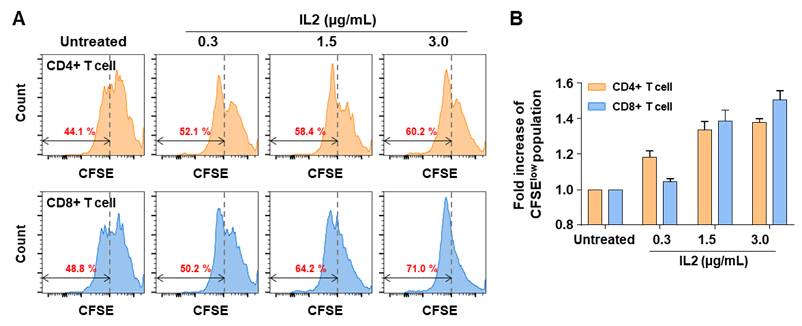


**Fig. S10. In vitro T cell expansion following treatment with IL2 at various concentrations.**

(A) Naïve T cells were stained with CFSE dye, and the proliferation of CD4⁺ and CD8⁺ T cells was measured by flow cytometry. (B) The fold increase in the CFSElow population in each group was quantified (*n =* 5).


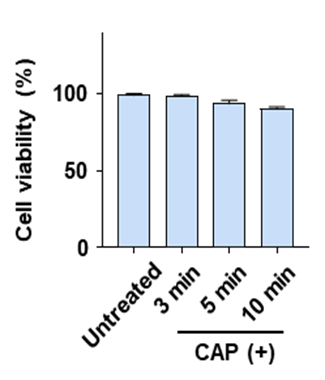


**Fig. S11. Cell viability following various durations of CAP treatment.**

T cell viability was measured after 1 h of incubation with CAP-treated media under different conditions. Statistical data are presented as mean ± SD (*n =* 3; n.s., not significant; ***p < 0.001).


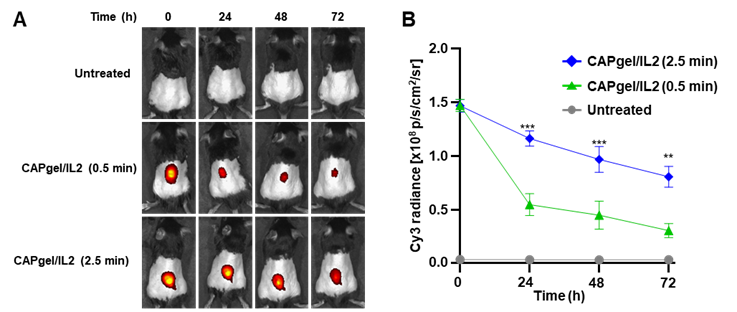


**Fig. S12. In vivo retention of CAPgel/IL2 by varying CAP exposure time.**

Whole-body fluorescence imaging was performed at 0, 24, 48, and 72 h post-injection. (A) Representative fluorescence images of mice administered with CAPgel/IL2 (0.5 min CAP), or CAPgel/IL2 (2.5 min CAP). (B) Quantification of fluorescence intensity showing the clearance of hydrogel after CAP irradiation.


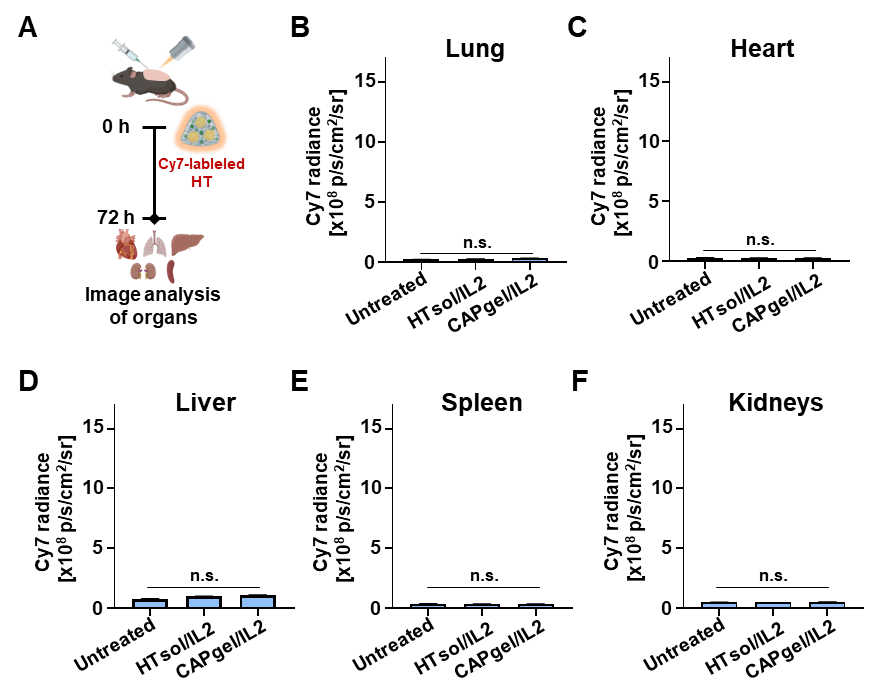


**Fig. S13. Distribution of Cy7-labeled HT to organs.**

(A) The schedule for the organ distribution study is illustrated. Cy7-labeled HTsol/IL2 was subcutaneously injected into the shaved dorsal skin and irradiated with CAP. At 72 h post-administration, organs including the lungs, heart, liver, spleen, and kidneys, were extracted for ex vivo imaging using the IVIS Spectrum instrument. (B-F) The quantified fluorescence intensities are presented for the lungs (B), heart (C), liver (D), spleen (E), and kidneys (F). All statistical data are presented as mean ± SD (*n =* 5; n.s., not significant).


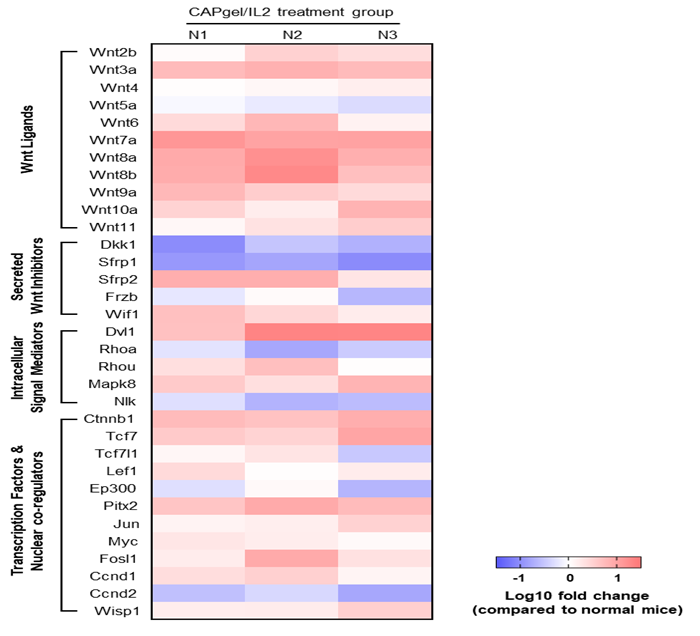


**Fig. S14. Gene expression profiles of Wnt signaling pathways in DPCs.**

DPCs were isolated from the dorsal skin of 7-week-old mice 15 days after CAPgel/IL2 treatment. Wnt signaling profiles were analyzed, and expression levels were normalized to GAPDH. Data are shown as a heatmap relative to untreated controls (*n =* 3).


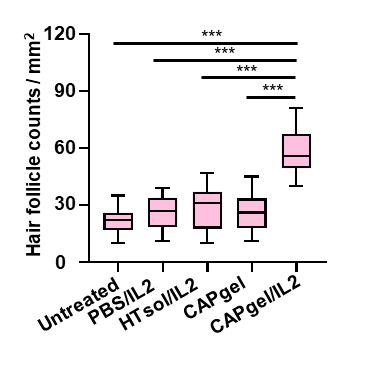


**Fig. S15. Hair follicle counts in skin tissue samples following various treatments.**

H&E-stained skin sections were analyzed to evaluate multiple parameters of hair follicle regeneration across treatment groups. Follicle counts (per mm² of skin within a depth of 1300 µm) were determined to assess the number of hair follicles.


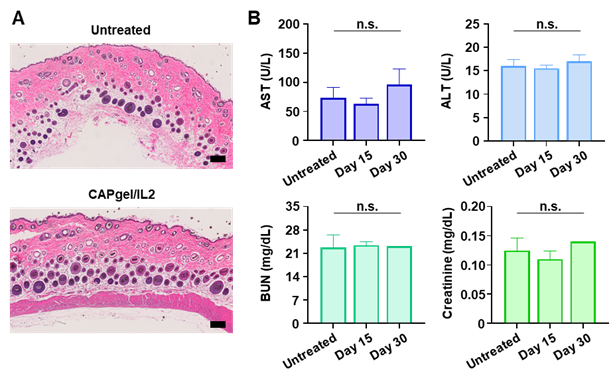


**Fig. S16. Tissue histology and serum biochemical markers over 30 days after CAPgel/IL2 treatment.**

(A) Representative H&E-stained skin sections were obtained from dorsal sites at day 30 after CAPgel/IL2 treatment (scale bar: 200 μm). (B) Serum levels of AST, ALT, BUN, and creatinine were measured. All statistical data are presented as mean ± SD (*n =* 5; n.s., not significant).


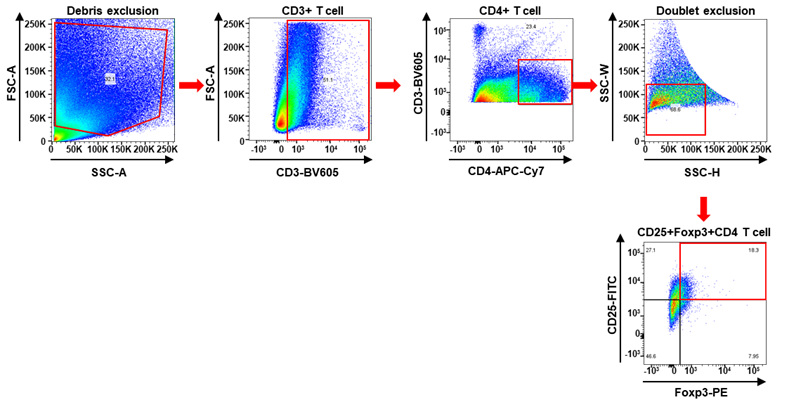


**Fig. S17. Gating strategy for in vivo CD25⁺Foxp3⁺ Treg cells FACS analysis.**

CD25⁺Foxp3⁺ Treg cells shown in Fig. 8H were gated from single suspensions of CD3+CD4⁺ T cells.


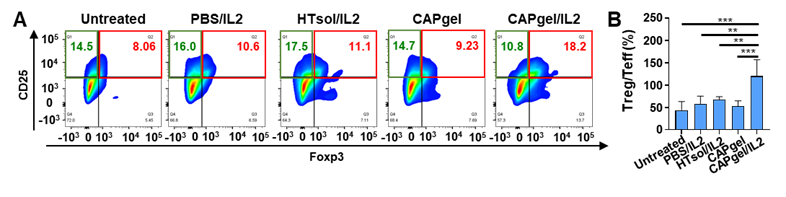


**Fig. S18. Flow cytometry of Treg and Teff following CAPgel/IL2 treatment.**

(A) Representative flow cytometry plots show CD25 and Foxp3 expression in CD4⁺ T cells from skin samples. (B) Tregs (CD25⁺Foxp3⁺) and activated effector T cells (Teffs, CD25⁺Foxp3⁻) were quantified and expressed as absolute percentages and as a Treg-to-Teff ratio (*n =* 4). All data are presented as mean ± SD; **p < 0.01, ***p < 0.001.
